# Properties, Genetics and Innate Immune Function of the Cuticle in Egg-Laying Species

**DOI:** 10.3389/fimmu.2022.838525

**Published:** 2022-02-25

**Authors:** Garima Kulshreshtha, Liliana D’Alba, Ian C. Dunn, Sophie Rehault-Godbert, Alejandro B. Rodriguez-Navarro, Maxwell T. Hincke

**Affiliations:** ^1^ Department of Cellular and Molecular Medicine, Faculty of Medicine, University of Ottawa, Ottawa, ON, Canada; ^2^ Evolutionary Ecology, Naturalis Biodiversity Center, Leiden, Netherlands; ^3^ The Roslin Institute and Royal (Dick) School of Veterinary Studies, University of Edinburgh, Edinburgh, United Kingdom; ^4^ INRAE, Université de Tours, BOA, Nouzilly, France; ^5^ Departamento de Mineralogia y Petrologia, Campus de Fuentenueva, Universidad de Granada, Granada, Spain; ^6^ Department of Innovation in Medical Education, Faculty of Medicine, University of Ottawa, Ottawa, ON, Canada

**Keywords:** eggshell cuticle, egg-laying birds, bacterial pathogens, food safety, microbiome, genetics, evolution

## Abstract

Cleidoic eggs possess very efficient and orchestrated systems to protect the embryo from external microbes until hatch. The cuticle is a proteinaceous layer on the shell surface in many bird and some reptile species. An intact cuticle forms a pore plug to occlude respiratory pores and is an effective physical and chemical barrier against microbial penetration. The interior of the egg is assumed to be normally sterile, while the outer eggshell cuticle hosts microbes. The diversity of the eggshell microbiome is derived from both maternal microbiota and those of the nesting environment. The surface characteristics of the egg, outer moisture layer and the presence of antimicrobial molecules composing the cuticle dictate constituents of the microbial communities on the eggshell surface. The avian cuticle affects eggshell wettability, water vapor conductance and regulates ultraviolet reflectance in various ground-nesting species; moreover, its composition, thickness and degree of coverage are dependent on species, hen age, and physiological stressors. Studies in domestic avian species have demonstrated that changes in the cuticle affect the food safety of eggs with respect to the risk of contamination by bacterial pathogens such as *Salmonella* and *Escherichia coli*. Moreover, preventing contamination of internal egg components is crucial to optimize hatching success in bird species. In chickens there is moderate heritability (38%) of cuticle deposition with a potential for genetic improvement. However, much less is known about other bird or reptile cuticles. This review synthesizes current knowledge of eggshell cuticle and provides insight into its evolution in the clade reptilia. The origin, composition and regulation of the eggshell microbiome and the potential function of the cuticle as the first barrier of egg defense are discussed in detail. We evaluate how changes in the cuticle affect the food safety of table eggs and vertical transmission of pathogens in the production chain with respect to the risk of contamination. Thus, this review provides insight into the physiological and microbiological characteristics of eggshell cuticle in relation to its protective function (innate immunity) in egg-laying birds and reptiles.

## 1 Introduction

**Figure 1:**
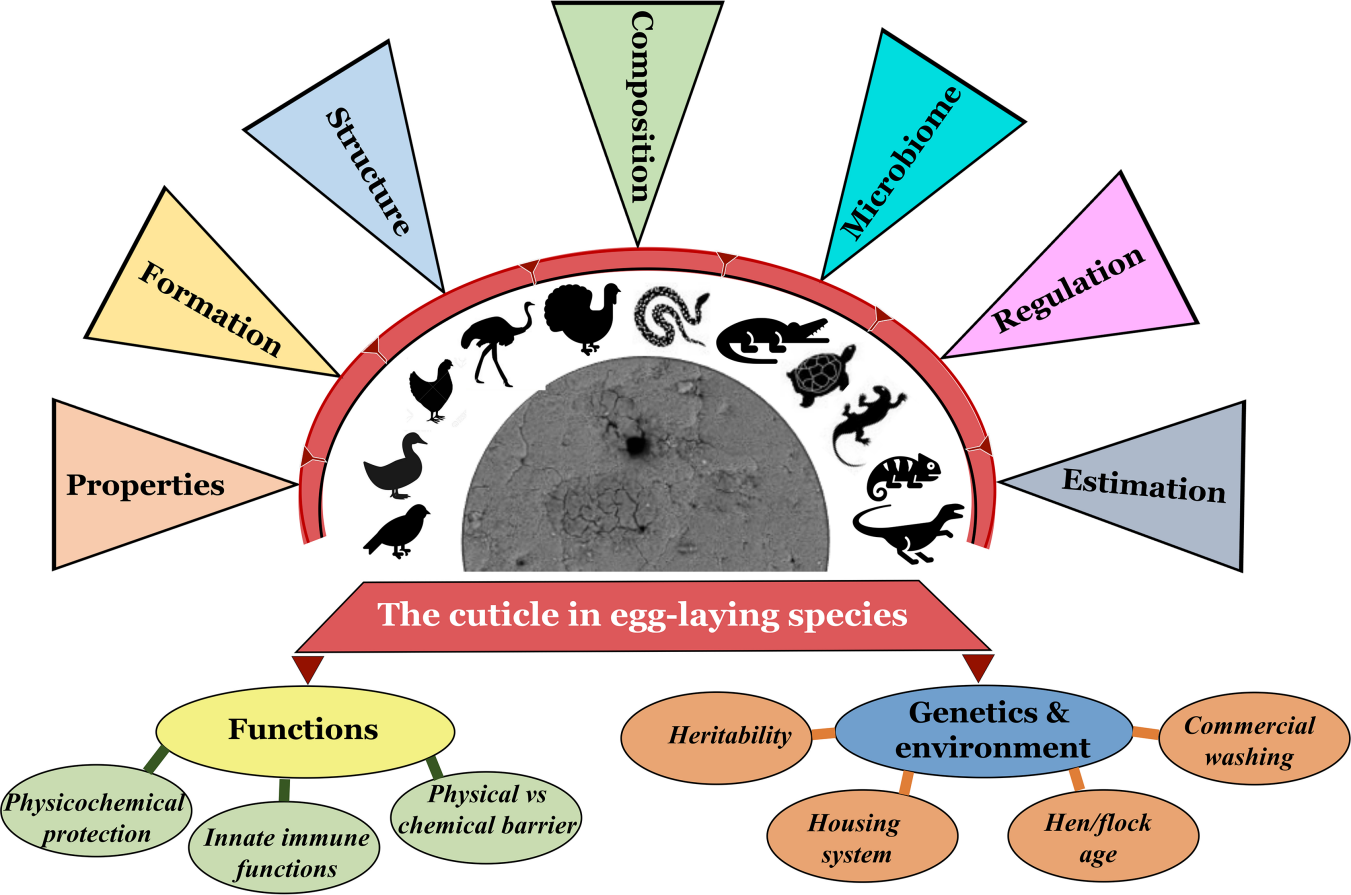
Graphical abstract navigating/cataloging the contents of this review article (Original figure by GK).

**Figure 2:**
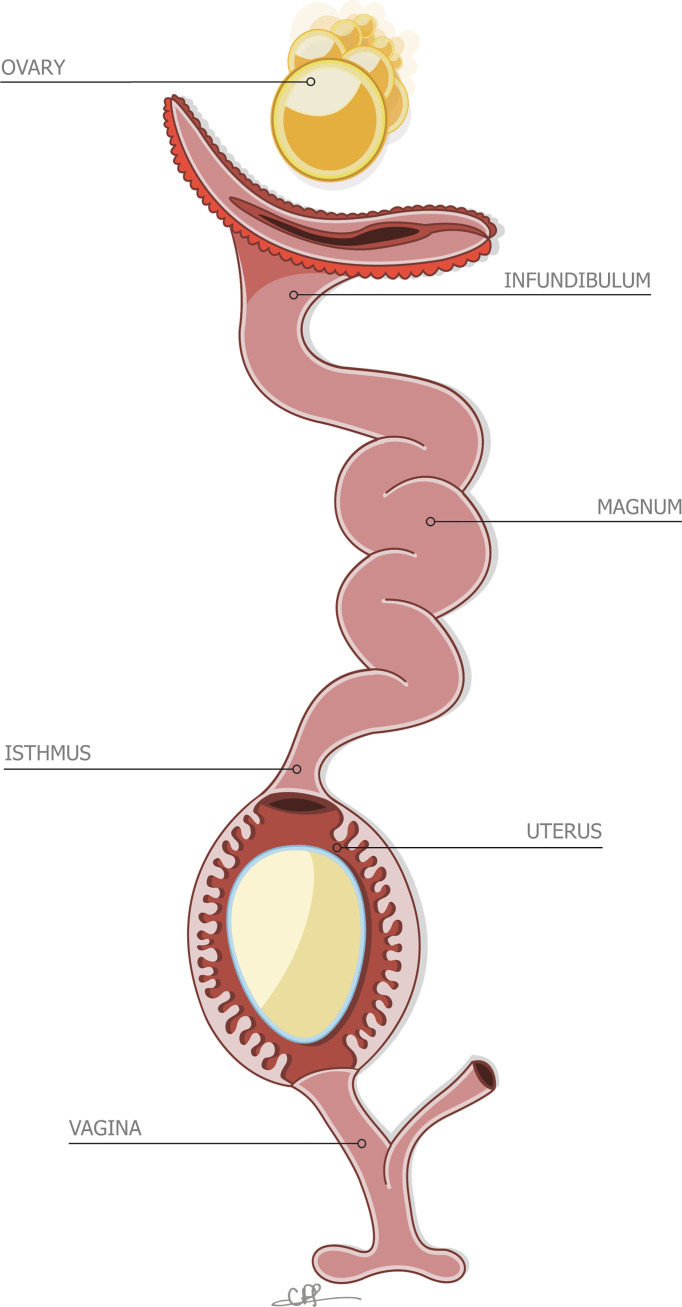
Stylized depiction of the reproductive system of the hen, containing an incomplete egg in the uterus. Reprinted from Front Bioscience., Vol. 17, Issue 1, Hincke et al., The eggshell: structure, composition and mineralization, 1266-1280, 2012, with permission from Frontiers.

**Figure 3:**
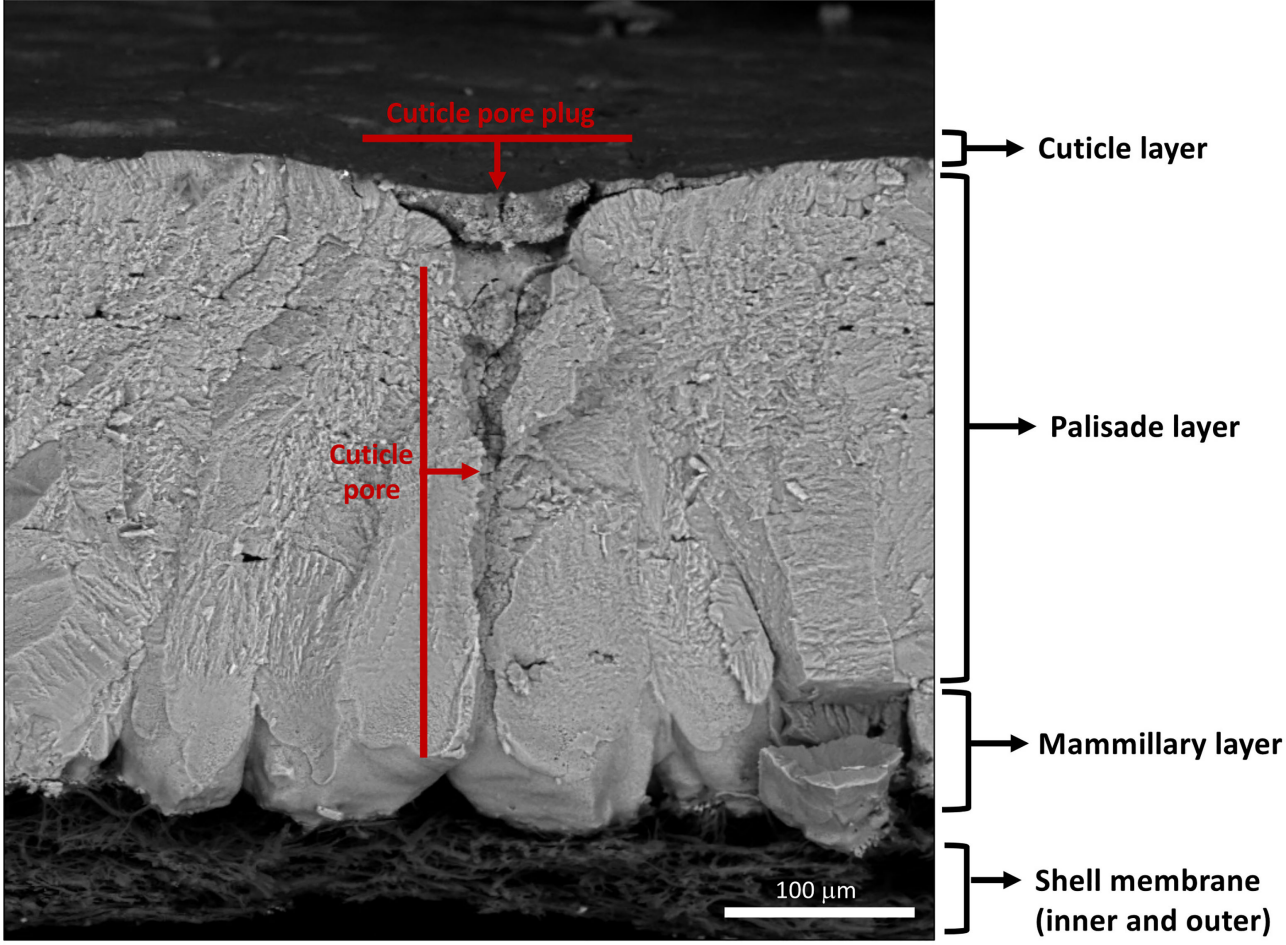
Scanning electron micrograph of cross-fractured eggshell showing different layers: cuticle, palisade layer, mammillary layer with associated inner and outer shell membranes and full-length respiratory pore with pore plug. (Original image by GK).

The cleidoic (rigid-walled) egg is a complete source of nutrients for embryonic development (1, 2). The egg contains molecules/substances with biological functions and activities such as antimicrobial, antioxidant, and immunomodulating, which highlight the nutritional importance of eggs and their components. Unfertilized eggs from species such as domestic chicken (*Gallus domesticus*), Guinea fowl (*Numida melleagris*), Quail (*Coturnix coturnix japonica*), Duck (*Anas platyhyncha*), Pigeon (*Columbia livia*) and Turkey (*Melleagris gallopavo*) are commercially produced for human consumption (3). Among these, chicken eggs are most commonly consumed as an inexpensive source of proteins in the human diet (1, 2). The egg contents are protected by a relatively impervious eggshell, a complex multifunctional bioceramic composed mainly of calcium carbonate (4, 5). The eggshell mineral layers are deposited/secreted sequentially during passage of the egg through specialized regions of the oviduct (6–8). From inside to outside, the chicken (*Gallus gallus*) eggshell is composed of highly ordered and distinct layers with variable thickness: the inner and outer eggshell membranes (~70μm), mammillary layer (~100μm), palisade layer (~300μm), vertical crystal layer (~3-8μm) and the cuticle (~0.5-12μm) (9–15) (**Figures 1**–**3**). The calcified eggshell is perforated by a large number of pores that permit exchange of water and gas which is essential for the developing embryo. However, the pores also allow bacterial pathogen ingress which can contaminate the egg contents (16–18). To avoid trans-shell contamination, the eggshell surface is coated by a thin and transparent organic layer, the cuticle, which plugs the pore openings in order to prevent microbial entry (17, 19, 20). Moreover, macromolecular components of the cuticle such as glycoproteins, polysaccharides, lipids, and abundant antibacterial proteins (e.g., lysozyme C, ovotransferrin, ovocalyxin-32 (OCX-32), ovocleidin-17(OC-17), constitute the basis for its antimicrobial activity (20–27). Thus, the cuticle provides both physical (5, 17, 28, 29) and chemical barriers against microbial aggression (24–26, 30–33). These barriers are critical for successful reproduction in egg-laying species, and moreover, serve to maintain food safety of the nutritious table egg for human consumption (34). Furthermore, it has been demonstrated that eggs with no cuticle or partially removed cuticle become more susceptible to bacterial contamination (27, 29, 35–37). The surface characteristics of the egg, outer moisture layer and the presence of antimicrobial molecules composing the cuticle regulate microbial communities on the eggshell surface (38–40). It has been suggested that the cuticle commensal microbes derived from both maternal microbiota and the nesting environment also participate in egg defense against pathogens, and this proposal will be explored at length in this review.

The cuticle fulfills a variety of diverse roles. Much knowledge of eggshell cuticle has been obtained from studies utilizing the chicken egg. However, much less is known about reptile cuticles. Structure of eggshell and its cuticle have evolved in response to diverse embryonic requirements and challenges, including protection from microbial infection, nest flooding, and exposure to solar radiation (41–43). This review describes detailed information on the eggshell cuticle and provides an insight into its evolution in the clade reptilia. We summarize function/importance of cuticle across diverse taxa in response to environment/selection pressure due to divergent evolutionary processes in eggs of bird and other species. Origin, composition and regulation of eggshell microbiome and the cuticle’s potential function as the first barrier of egg defense are discussed. Current consumer demand for poultry eggs has expanded from chicken and duck eggs to other more ‘exotic’ species such as quail, turkey, pigeon, and ostrich eggs. Hence, we discuss how changes in the cuticle affect the food safety of eggs with respect to the risk of adherence and trans-shell penetration by bacterial pathogens such as *Salmonella* and *E. coli*. Thus, this review will provide insight into the physiological and microbiological characteristics of eggshell cuticle in relation to its protective function (innate immunity) in egg-laying birds and reptiles (**Figure 1**).

## 2 Properties of the Cuticle Across Egg Laying Species

### 2.1 Cuticle in Reptile Eggs

**Figure 4:**
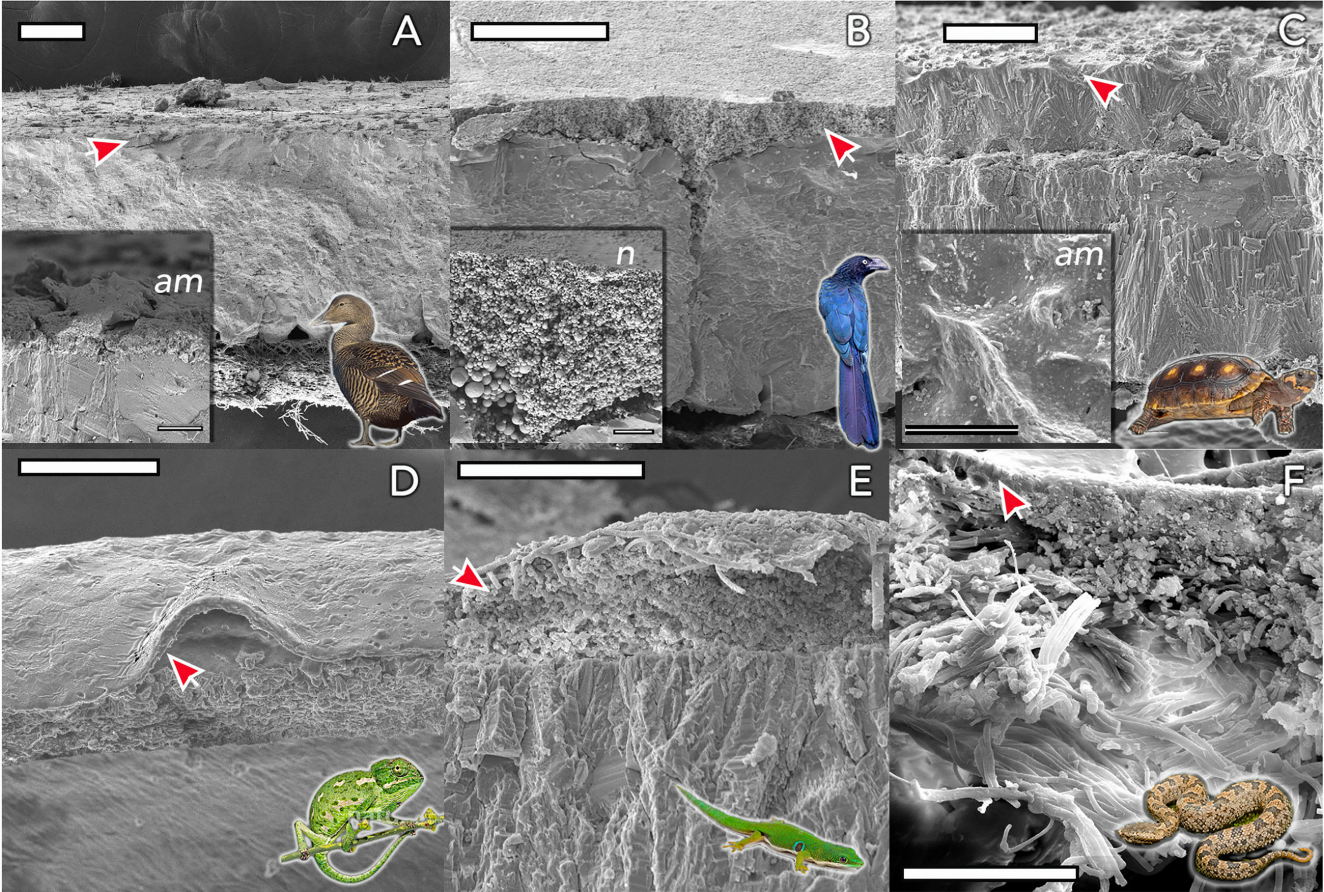
Eggshell ultrastructure in representative species of amniote vertebrates. The eggshell cuticle (arrows) is the most superficial layer covering the calcified or fibrous shell. **(A)** Common eider (*Somateria mollissima)*; **(B)** Great ani (Crotophaga major); **(C)** red-footed tortoise (*Chelonoidis carbonarius*); **(D)** Mediterranean chameleon (*Chamaeleo chamaeleon);* **(E)** Madagascar day-gecko (*Phelsuma madagascariensis*); **(F)** Mountain pit viper (*Ovophis monticola*). Insets show detail of representative amorphous (*am*) and nanostructured (*n*; nanospheres) cuticles. Scale bars: **(A, B, D)** -100 μm; **(C)** - 200 μm; **(E, F)** - 50 μm. (Original figure by LDA).

The cuticle in reptile eggshell has not been formally defined and often, diverse nomenclature is used to describe this layer e.g. *covering layer* (44, 45); *cover* (46); *cuticle* (15, 47). It also has been argued that the reptilian cuticle is not equivalent to the cuticle present on avian eggshells (48); however, a formal comparison has never been done. To date, few reports exist on the cuticle in non-avian eggs. This missing information likely results from the general scarcity of studies about egg formation and composition in non-avian reptiles. In reptile species, cuticle is more commonly observed on calcified eggshells of some turtles and geckos, and less often on soft shells as those of snakes and lizards. With the exception of crocodiles (48), cuticle has been found on eggs of all reptile groups including turtles, snakes, lizards and geckos (**Table 1**), and shows large variation in structure (**Figure 4**). In crocodiles, organic material was observed plugging the pores of American alligator (*Alligator mississippiensis*) eggs (48). This consisted of crystalline nanospheres, which covered the surface of the shell for a few days after oviposition, but then disappeared, perhaps due to acidic erosion inside the nest. Despite the similarities with the pore plugs formed by the cuticle of avian eggs, it has been argued that this material should not be considered a true cuticle (48). In other reptiles, cuticles vary in thickness from 2 to 50 um (**Table 1**) and can appear as a homogeneous amorphous layer, a mixed layer containing crystals or spherulitic granules (e.g., *Sceloporus virgatus*; **Figure 4**) (52).

**Table 1:** Published reports of eggshell cuticle in non-avian reptiles and information on cuticle composition and specific nesting habitat.

Species	Cuticle Composition	Thickness	Nesting Habitat	Reference
Crocodiles	–	–		–
Turtles				
Red-headed Amazon Side-necked Turtle *(Podocnemis erythrocephala)*	–	~2 μm	Tropical rivers	44
Red-footed Tortoise *(Chelonoides carbonaria)*	Protein fibrils, calcite crystals	26-30 μm	Tropical savanna	49, 50
(Indian) Star Tortoise *Geochelone elegans*	“	32 μm	Scrublands, during monsoon rains	49
Burmese Star Tortoise *(Geochelone Platynota)*	“	52 μm	Xerophytic	49
Squamates	Soluble proteins			44
Desert Agama *(Trapelus mutabilis)*	–	20 μm	Desert	44
Brown Basilisk *(Basiliscus vittatus)*	–	2 μm	Deciduous tropical forest	44
Sand Lizard *(Lacerta agilis)*	–	6 μm	Dry grassland, heathland	44
Argentine Black and White Tegu *(Salvator merianae)*	Glycosaminoglycans	6 μm	Tropical savanna	51
Madagascar Day Gecko *(Phelsuma madagascariensis)*	–	16 μm	Rainforest	44
Madagascar Giant Day Gecko *(Phelsuma grandis)*	–	7 μm	Subtropical forest	50
Kilimanjaro two-horned Chameleon *(Chamaleo fischeri tavetanus)*	–	15 μm	Tropical savanna	44
Senegal Chameleon *(Chamaeleo senegalensis)*	–	~15 μm	Tropical savanna	44
Gemeines Chamaleon *(Chamaeleo chamaeleon)*	–	11 μm	Dry woodland	50
Palestine Viper *(Vipera palaestinae)*	–	~ 2 μm	Mediterranean coastal plains, shrubland	44
Eastern Racer *(Coluber constrictor)*	–		Temperate grassland	45
Striped Plateau Lizard *(Sceloporus virgatus)*	–		Scrub forest	52
Fence Lizard *(Sceloporus undulatus hyancinthinus)*	Unknown organic material, (non-calcified)	2 μm	Temperate forest	46
Clark’s Spiny Lizard *(Sceloporus clarkia)*	“	2 μm	Temperate shrublands	46
Light bellied Bunch Grass Lizard *(Sceloporus scalaris)*	“	2 μm	Dry grassland, scrubland	46
Tokay Gecko *(Gecko gecko)*	Organic material, calcium carbonate Proteins + high concentrations of S and Mg, calcite spherical granules.		Rocky grassland and desert	44
Stumpff’s Ground Gecko *(Paroedura stumpfii)*	“		Tropical forest	44
Pictus Ground Gecko *(Paroedura pictus)*	“		Tropical forest	44
Madagascar Giant Day Gecko *(Phelsuma grandis)*	“		Tropical forest	44
Carter’s Rock Gecko *(Pristurus carteri)*	–		Hot and arid plains	50
Sinai Fan-fingered Gecko *(Ptyodactylus guttatus)*	–		Rocky grassland and desert	50

Eggshell cuticles have also been identified in fossilized eggs of a non-avian theropod dinosaur (53) and enantiornithines (54, 55), an extinct group of avialans from the Mesozoic. One remarkably preserved enantiornithine egg was discovered as a single unlaid egg, within the abdominothoracic cavity of the mother. It exhibits a distinctive cuticle layer enriched in phosphorous and consisting of spherical nanostructures of calcium phosphate (55) similar to those observed in modern bird cuticles. This discovery supports in favor of the presence of cuticle nanospheres in the common ancestor of birds.

### 2.2 Formation of Egg in the Avian Reproductive Tract

Due to its commercial importance, most studies of egg formation have focused on the chicken (*Gallus gallus*), and consequently we know more about the reproductive physiology of this bird than any other avian species. Therefore, here we have highlighted the deposition of the cuticle in the chicken reproductive system. Egg formation is initiated by ovulation (release of an ovarian follicle into the proximal oviduct), and the forming egg sequentially acquires its compartments as it transits different segments (**Figure 2**). Following mineralization of the calcified shell in the uterus (shell gland pouch), the cuticle layer is deposited on its surface. The cuticle constituents are secreted by non-ciliated secretory cells during the last 2 h before egg expulsion (oviposition) (4, 56, 57). The normal endocrine events which regulate ovulation and oviposition are necessary for deposition of the cuticle (57). Uterine transcriptomic analysis in chickens laying eggs with different degrees of cuticle deposition suggests that at least two categories of genes are responsible for controlling the production of cuticle (58). Cuticle deposition and oviposition are mediated by both clock genes, which regulate the timing of cellular events to ensure that cells respond appropriately to their environment, and immediate early genes, which are critical in the activation of cellular processes by external factors (58). The lack of differences in gene expression between the uterus of hens laying eggs with the best and worst cuticle deposition indicated that the genetic variability of the trait could lie outside the oviduct (58). In another recent study, transcriptomic analysis has demonstrated that the physiological state of the uterus regulates eggshell quality and egg ultrastructure. Specific genes (*PTGDS*, *PLCG2*, *ADM* and *PRLR)* are predicted to play a critical role in cuticle deposition by modulating uterine secretion rhythm and function (2).

Cuticle deposition is distinct from other events in egg formation. Since termination of shell mineralization occurs before cuticle deposition, the cuticle is not contiguous with the organic matrix of the eggshell (27), instead it is a specific secretion which is distinct from other events in eggshell calcification (56). Avian eggs exhibit variability in shell coloration/pigmentation due to the presence of blue-green biliverdin and red-brown protoporphyrin pigments deposited into the outer surface of a developing egg in the shell gland (59); however, no direct correlation has been established between deposition of shell pigment and cuticle formation. Most of the shell pigment is located in the outer calcified layers, with only 13–20% found in the cuticle and, although pigment deposition and cuticle deposition are temporally close (60, 61), pigment deposition occurs earlier; it is almost complete an hour before the expected time of oviposition. According to genetic studies, there is no connection between the genes controlling shell pigment deposition and cuticle formation, and neither event is dependent on the other (56). In at least one avian species (tinamou), the glossy appearance of the eggshell is produced by an extremely smooth cuticle, composed of calcium carbonate, calcium phosphate, proteins and pigments (62).

There have been several anatomical investigations of the oviductal morphology in reptiles (63–65). However, these studies have not provided detailed information about eggshell formation. Unlike birds, reptiles possess an oviduct undifferentiated into separate anatomical regions. The exception to this rule are crocodilians, which have an oviduct demarcated into six regions, homologous to that of birds (66). Thus, in chelonians and squamates, the oviduct is not specialized for the production of different eggshell components and the proteinaceous membrane and calcified shell are both deposited in the homogeneous uterus (64). Furthermore, the entire process from ovulation to shell deposition is much longer in reptiles compared to the average 24 hours in the fowl (67). Deposition of cuticle may initiate several days after ovulation, concurrent with a change in shape of the fibrous shell membrane and its adoption of peaks and valleys. It has been hypothesized that the superficial deposition of minerals and organic matter might be initiated, at least in part, by the reorganization of the shell fibers and subtle chemical changes in the oviduct (67).

### 2.3 Structural Organization

The term “cuticle” a distinct layer on the outer surface of the calcified or fibrous shell, of organic or mixed composition, and of oviductal origin. It is also defined as shell accessory material (SAM) on external surface of eggshell in some studies on bird species including Mandarin duck (*Aix galericulata*), domestic turkey (*Meleagris gallopavo*) and goose (*Anser anser*) (68, 69). The cuticle of chicken eggs is a relatively thin layer with variable thickness (0.5-12 μm), with patchy and uneven distribution on the eggshell surface (20, 70, 71). It is more complete in freshly laid eggs and when dry, has a glossy appearance (20). Variable cuticle thickness is observed in eggs of many other avian species (15). For example, the approximate thickness of the cuticle layer varies considerably between the White Pelican (*Pelecanus onocrotalus*) (130 μm), Japanese quail (*Coturnix japonica*) (10 μm), Greater Flamingo (*Phoenicopterus ruber roseus*) (110 μm), and Humboldt Penguin (*Spheniscus humboldti*) (45 μm) (15). On the other hand, the cuticle is absent in eggs from some clades such as parrots, petrels and pigeons (42). It has been proposed that environmental or reproductive selection pressures are responsible for divergent cuticle features in eggs of different species (42).

**Figure 5:**
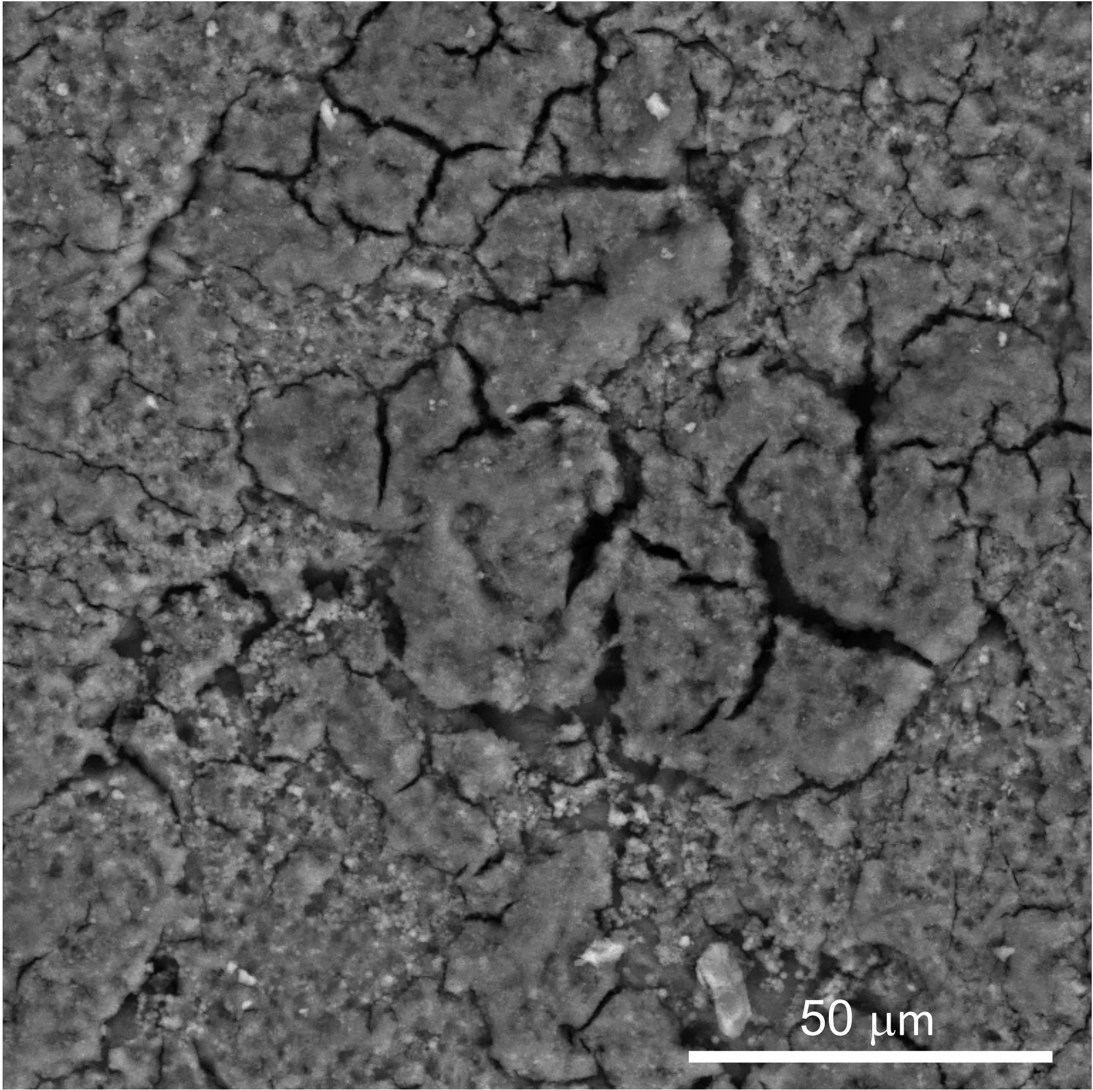
Scanning electron micrograph (SEM) image of outer surface of chicken eggshell cuticle at 1000X showing patchy distribution with cracks and fissures. (Original figure by GK).

**Figure 6:**
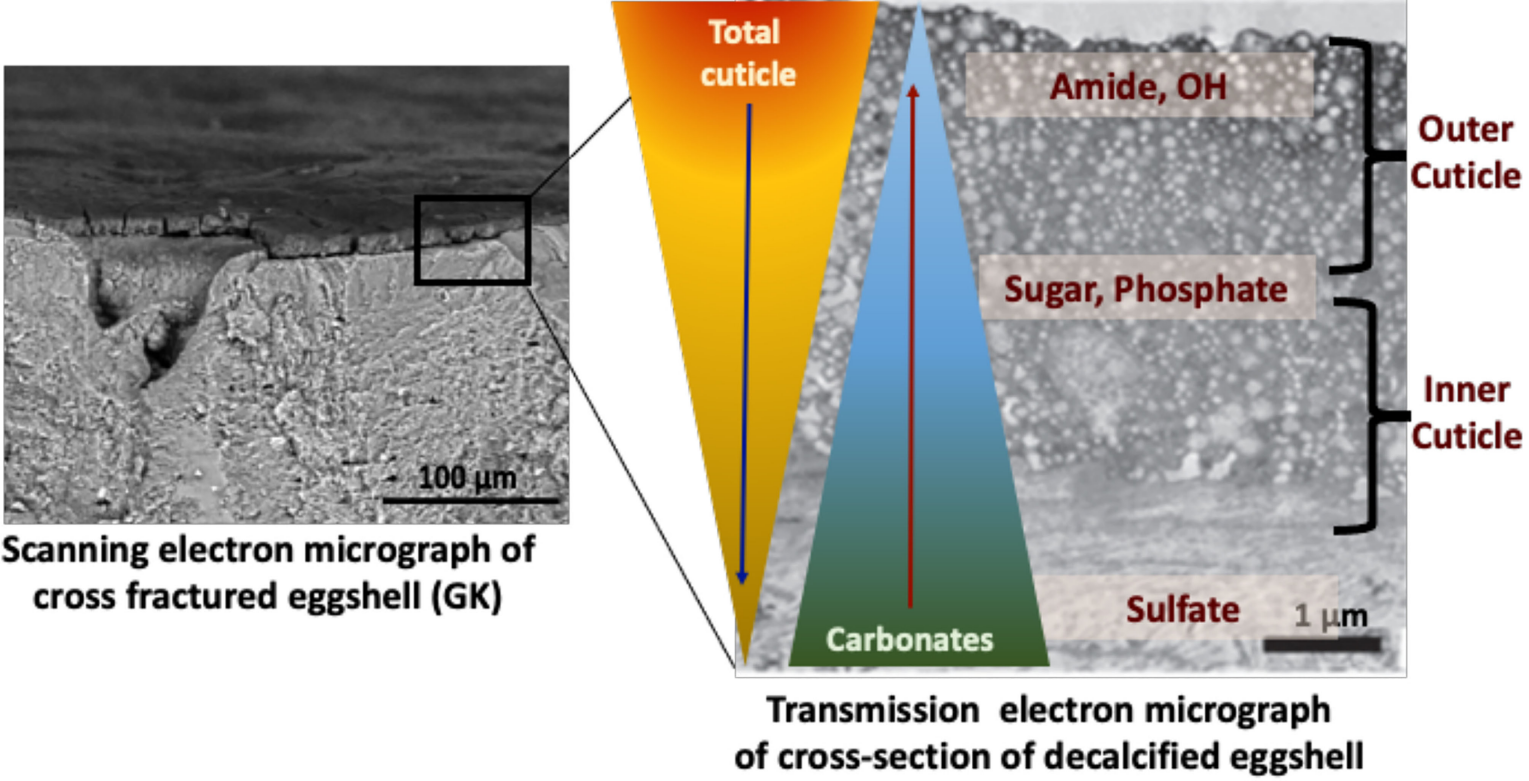
Proposed compositional gradient in the eggshell cuticle with predicted distribution of proteins, phosphoproteins, glycoproteins, and sulphated proteoglycans. Reprinted from Foods, Vol. 10, Issue 11, Kulshreshtha et al., Impact of different layer housing systems on eggshell cuticle quality and *Salmonella* adherence in table eggs, 2559, 2021, with permission from MPDI.

The structure and morphology of the cuticle can be evaluated by scanning and transmission electron microscopy (SEM, TEM) (**Figures 3**, **5** and **6**) (20). As the cuticle dries and hardens upon exposure to the environment, it displays micro-cracks and micro-fissures on its outer surface, as visualized by SEM (**Figure 5**) (15, 72, 73). The chicken cuticle is composed of two layers: inner (vesicular) and outer (non-vesicular) layers identified by TEM (**Figure 6**). The inner layer is composed of vesicles ranging from approximately 50 to 500 nm that are deposited during the final phase of eggshell calcification (termination) (20). Each vesicle is composed of a core and mantle with electron lucent and electron dense properties, respectively (74). The non-vesicular outer cuticle is more compact and homogenous; it is a water-insoluble layer with non-mineralized components (15). TEM imaging also reveals that cuticle thickness varies widely in chicken eggs, even being completely absent in some regions (20, 70).

In avian species, the inner cuticle is composed of vaterite crystals, but is organized as nanospheres on others (42). Many species lack nanospheres in their cuticle, but this nano structuring is hypothesized to have been present in eggs of the avian ancestor (42).

### 2.4 Composition of the Cuticle

Currently, our knowledge about composition of reptilian cuticles is very limited. Early studies showed that the cuticle in squamates (a monophyletic group comprising lizards, snakes and amphisbaenians) contains primarily neutral hydrophobic amino acids, soluble proteins, which vary greatly in composition, and negligible amounts of lipids (75). More recently, carboxylated carbohydrates and sulfated mucins have been reported in the cuticles of eggs of the Argentine black and white tegu (*Salvator merianae*) (49). The presence of phosphorous is a clear attribute of the cuticle layer in birds. The function of phosphorous is not entirely clear, although it has been suggested that the presence of phosphate terminates eggshell growth because it competes with carbonate for calcium during eggshell mineralization (49). However, in order to interpret the significance of phosphate/phosphorus in cuticle, it will be important to distinguish between inorganic phosphate (i.e. as hydroxyapatite (68) and that present in phosphorylated cuticle proteins such as OCX-32 (5, 7). Large concentrations of phosphorous have also been detected in the cuticles of non-avian reptiles (45, 76).

#### 2.4.1 Proteins and Peptides Constituents of the Cuticle

In chicken eggs, the non-vesicular outer cuticle layer is composed of proteins/glycoproteins (85-90%), carbohydrates (4-5%), lipids (2.5-3.5%) (20–23). These macromolecules play a vital role in both physical and chemical antimicrobial defense mechanisms to restrict the entry and growth of invading pathogens (24, 25, 27). The cuticle is mostly organic with high protein content (approaching 90%) and possessing a high content of glycine (9.3-15.2%), glutamic acid (10.7-18.7%), and tyrosine (7.1-9.0%) (22, 23). The average dry weight of the cuticle of a White Leghorn egg (60 grams) is approximately 12 mg (11). Cuticle can be partially removed from the shell by washing with water (>40°C) or by mechanical abrasion (11). Detergents and dilute acids are more effective in isolating/removing the cuticle from the shell surface (11, 77).

ATR-FTIR analysis of chemical components of the cuticle showed a strong negative correlation of cuticle protein signal with carbonate (eggshell) and a moderate positive correlation with sugars and phosphate, indicating a gradient in chemical composition of cuticle, with the outer part being richer in proteins and the inner part being abundant in glycoproteins, proteoglycans, polysaccharides and phosphate (34) (**Figure 6**).

### 2.5 Regulation of Synthesis, Composition, and Deposition of the Cuticle

All evidence points to the uterus (shell gland) as the oviduct segment where cuticle production and its deposition occurs (56, 78–80). It is observed that material accumulating in the epithelial and tubular gland cells of the chicken shell gland lining before oviposition, such as dermatan sulfate, has disappeared from these cells after oviposition (79). In quail, an unidentified protein found on the shell and in the shell gland also accumulates towards the end of egg formation and is largely depleted after oviposition (80). OCX-32, a significant component of the cuticle, disappeared from the shell gland secretory cells when an egg was laid normally, but not when the egg was laid prematurely without a cuticle (56). Furthermore, OCX-32-positive cells are not found in the vagina (56). Finally, a study in which eggs were removed from the oviduct just before oviposition also concluded that the cuticle was formed in the uterus (78).

Thus, there is strong evidence that the cuticle is deposited at the very end of egg formation, not least because it is the last layer of the egg structure. Previous studies on the relative timing of cuticle deposition and oviposition had only allowed an estimate to within 4 h pre-oviposition (80). A more recent study narrowed this interval, demonstrating that pigments, where present, were laid down an hour before oviposition and the cuticle secretion therefore occurred less than an hour in advance of oviposition (56). Even with synchronized oviposition, reducing the estimate of cuticle deposition in proximity to oviposition is difficult, due to natural variability in timing between individuals.

There is limited information on the control of cuticle deposition. Clearly, as we will see in a later section (4.2), genetics controls a relatively large part of the variance in cuticle deposition (25, 56); however, there is much less information concerning the importance and nature of non-genetic factors. Mild stress in hens, such as relocation to pens from cages, does reduce cuticle coverage, although the effect is relatively small (56). The effect of hen age remains controversial. In a longitudinal study, where up to 100 hens from both layer and broiler breeder lines were followed from peak of lay to 50 weeks of age, there was no statistically significant effect of hen age on cuticle coverage, although brown eggshell pigment was clearly reduced in older birds (81). Moreover, no change in cuticle deposition in hens up to 70 weeks of age was observed in a commercial flock of Leghorn hens laying white eggs (82). Another study using white eggs laid by Hy-Line CV22 hens observed no significant change in cuticle amide content between 16 and 70 weeks of hen age (20). On the other hand, there was a significant decrease in cuticle amide content in both white and brown eggs from Lohmann hens over 21 to 66 weeks of age (17). Therefore, the influence of hen age upon cuticle deposition remains unclear, but there may be distinctions between different lines of hens, egg color and possibly rearing/caging systems.

Moreover, physiological control of cuticle deposition is not well understood. Inducing premature oviposition of the egg with gonadotropin releasing hormone (GnRH) at the top of the endocrine cascade resulted in a normal cuticle after oviposition, whilst conversely the induction of oviposition by arginine vasotocin (AVT) or prostaglandin F2alpha (PGF2α) at the bottom of the endocrine cascade that induces oviposition does not (56). Thus, there are indications that a number of hormones or factors might induce cuticle secretion and deposition if they occur in the correct order and the most proximate endocrine signals on their own are not sufficient, but definitive evidence remains elusive (56).

### 2.6 Cuticle Estimation

It is intriguing that the standard measurements of eggshell quality, which are very important selection tools to improve production parameters in the poultry industry, do not include cuticle quality. Nonetheless, various methods have been evaluated to assess cuticle quality i.e. the amount or degree of coverage on the eggshell surface and chemical composition, including cuticle staining, Attenuated total reflection Fourier transform infrared spectrometry (ATR-FTIR), tryptophan fluorescence and eggshell surface contact angle (hydrophobicity).

#### 2.6.1 Estimation of Cuticle Quality by Staining

**Figure 7:**
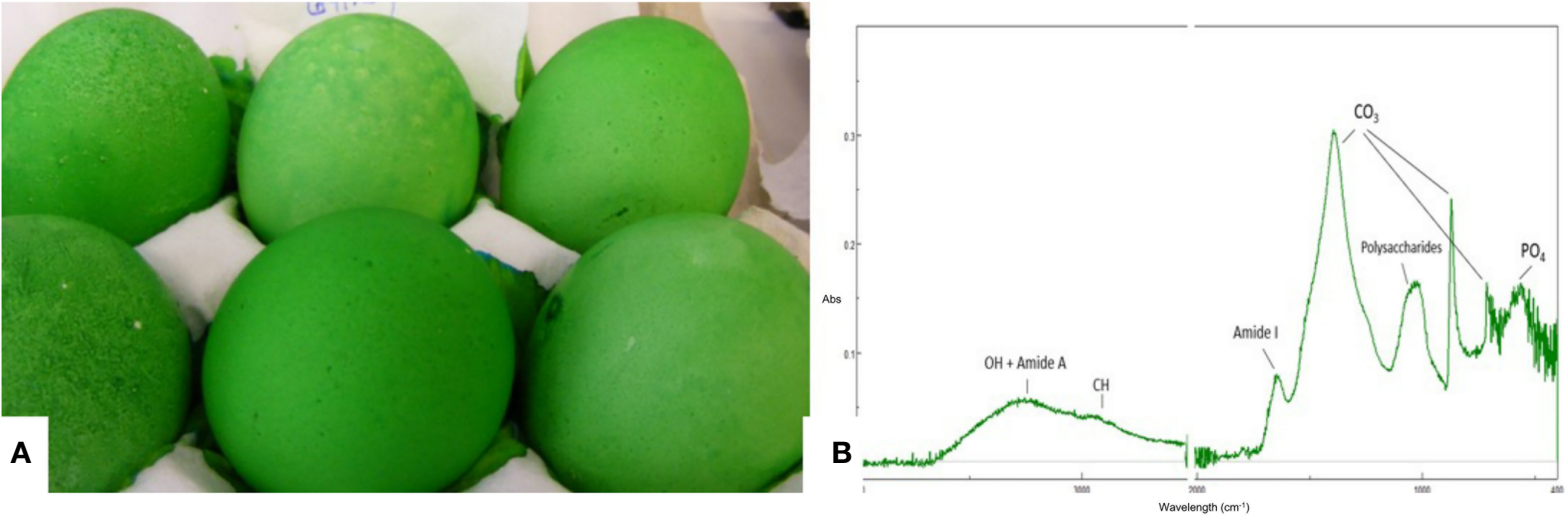
Characterization of the eggshell cuticle. **(A)** Eggs demonstrating a good degree of uniform staining with MST cuticle blue dye; **(B)** ATR-FTIR spectra of an eggshell surface showing the main IR bands from the cuticle and from the shell mineral. (Original figure by ARN).

Cuticle quality is traditionally assessed by staining intact eggs with specific dyes (i.e., Edicol pea green, MST cuticle blue) that have an affinity for cuticle proteins and color the eggshell green if this organic coating is present (**Figure 7**). This method can be easily applied to a large number of eggs and the amount of cuticle and degree of coverage can be scored visually based on a color scale. For a more quantitative estimation, color intensity can be measured with a color spectrophotometer to determine a parameter (ΔE_ab_) based on L*a*b* color space (27, 29, 36, 83). The degree of shell color intensity can be reliably measured with a Konica Minolta spectrophotometer at 650 nm. The amount of cuticle is estimated by the percent reflectance difference (Δ650 nm) of the egg before and after staining (27). A recent study used optical theory to improve the staining method in order to evaluate the cuticle quality of differentially colored eggs (84).

#### 2.6.2 Estimation of Cuticle Chemical Composition

Cuticle chemical composition affects its functionality. For instance, protein content determines the resistance of the cuticle against bacterial penetration (73). ATR-FTIR is a surface characterization technique that probes the amount and chemical composition of the cuticle. It is especially well suited for cuticle analysis as the ATR signal penetration depth is a few microns, comparable to the thickness of this layer (**Figure 7**) (20). The main chemical components of the cuticle (water, proteins, polysaccharides, lipids) and of the shell mineral (carbonate) contribute to the IR spectra absorption peaks associated with molecular groups of each component (e.g. O-H: water; amide: proteins; C-O: carbonates) (20). The relative intensity of each peak (i.e., amide) is proportional to the concentration of the associated component (i.e., proteins). On the other hand, as the cuticle thickness increases, the intensity of the protein and polysaccharide peaks increase, while the intensity of carbonate peaks from the underlying shell mineral decreases. The intensity of the OH band from water can be used to assess shell permeability. However, ATR-FTIR measurements, being local (probe diameter of about 2 mm) does not provide information about the overall degree of cuticle coverage on the egg surface (20).

#### 2.6.3 Tryptophan Fluorescence Intensity

Simple measurements of cuticle quantity would be advantageous for research and genetic selection. It was reasonable to predict that the intrinsic UV fluorescence of tryptophan content of the cuticle proteins would be a good proxy for cuticle content. However, this measurement was not found to be useful for assessment of cuticle because the emitted fluorescence at 330nm is quenched by other components of the shell, including protoporphyrin pigment and the proteins of the shell matrix, with contributions from the matrix fluorescence further complicating evaluation (85). Therefore, although this measurement had relatively good heritability, it showed no genetic correlation with measurement of cuticle by MST Cuticle Blue staining (85).

#### 2.6.4 Contact Angle (Hydrophobicity)

The outer surface of some avian species has evolved hydrophobic surfaces to prevent water entry in order to resist bacterial contamination, as the surface wettability has an impact on primary bacterial adhesion. Measurement of contact angle to determine surface hydrophobicity of the outer surface of individual eggs has been proposed for assessment of the cuticle quality (34). A correlation between hydrophobicity (contact angle) with cuticle chemical components determined by infrared spectroscopy (ATR-FTIR: positive correlation) and with bacterial cell count (negative correlation) on the eggshell surface, provide evidence that contact angle could be an accurate measure of cuticle quality (hydrophobicity) which is important for food safety of the table eggs (34). This method involves precisely placing a droplet of deionized water on the egg surface and accurately/rapidly measuring contact angle by image acquisition and analysis, in order to determine cuticle quality. Major advantages of this method are: cost-effective, rapid, non-invasive, and non-destructive. This method has the potential to be utilized at commercial egg grading and hatchery systems to evaluate cuticle quality with high throughput.

### 2.7 Eggshell Microbiome

The outer eggshell cuticle hosts microbes but the nature of the endogenous egg microbiome remains unclear. For example, it is controversial whether the interior of the egg is sterile, although this is a frequent assumption. There is a distinction between the two main mechanisms of egg contamination: vertical transmission (transovarian route) during eggshell formation in the oviduct is contrasted with horizontal transmission (trans-shell penetration) that occurs following oviposition (86). Notably, the cuticle hosts commensal microbes from maternal origin that interact with environmental microbes. The combination from these microbial origins will form the cuticle/eggshell microbiota, which is hypothesized to participate in egg defense against harmful pathogens. The abundance and the phylogenetic diversity of the cuticle microbiota depends on many factors including wettability (presence of moisturizing factors, 3.1), surface structure (2.3) and biochemistry of the eggshell (2.4; 3.2), in addition to the climate, nesting environment (nature of the nest material) and parental care (peculiarities of incubation conditions and parental presence). Hence, the composition of the egg microbiome may vary across large ecological scales and result from multifactorial variables.

Once the cuticle is exposed to microbes, including both Gram-positive and Gram-negative strains, the probability of trans-shell infection post-oviposition is affected by local environmental conditions (especially when high temperature meets high relative humidity), the exposure period and the nature of eggshell microbiota (38–40). The subsequent contamination of the internal egg components by Gram-negative microbes, as frequently observed, is then dependent on the capacity for bacteria to penetrate the eggshell (motile capacity of non-clustering bacteria, moisture), followed by their resistance to the egg white antimicrobial activities, high pH and viscous nature (36)

Thus, the eggshell microbiota is an inheritance from both the maternal microbiota and the environment. At laying time, the egg surface is moist but will progressively dehydrate before and during incubation. The surface characteristics of the egg, the loss of the moisture layer, the availability of nutrients, and the presence of antimicrobial molecules (3.2) are thought to dictate the phylogenetic composition of the eggshell microbiota that will survive on the eggshell surface during incubation. All these interacting factors are crucial to influence the probability of trans-shell contamination of the egg. However, the dominant factor that is invariably associated with hatching failure in both wild and domestic species, is pathogen pressure from the environment and the subsequent bacterial load on the eggshell (38, 39, 87–91). Noticeably, the eggs of different avian species can support different bacterial densities and diversity, but hatching success remains similar between avian species, independent of incubation specificities (parental or remotely from parents). Such an observation suggests a complex but finely balanced regulation between all intrinsic and external components of egg incubation, in order to maintain hatching success at similar levels in all species (88). It also supposes adaptive mechanisms that have evolved in response to differences in climate and habitat.

#### 2.7.1 Vertical Transmission

As mentioned previously, vertical transmission refers to direct contamination of the yolk, albumen, shell membranes, and eggshell before oviposition, originating from the infection of reproductive tract with pathogenic microbes. After ovulation and capture of the follicle by the proximal oviduct, the forming egg will pass through several reproductive segments, from the infundibulum, magnum, isthmus, uterus, vagina, and finally expulsion *via* the cloaca (2.2), where the egg encounters caecal secretions from the gut tract (**Figure 2**). In chicken species, 21 bacterial genera were shown to be common between the maternal reproductive tract/cloaca and the descendant eggshell (92). Oviductal regions (infundibulum, magnum, uterus and vagina) exhibited comparable microbiota composition, with Proteobacteria and Firmicutes being the dominant microbial phyla (93) followed by Bacteroidetes (infundibulum and magnum), Actinobacteria (magnum) and Fusobacteria (vagina). The similarity in composition and the gradual increase in diversity from the infundibulum to the vagina, suggests a microbiota continuum along the reproductive tract. Genera identified in the reproductive tract are also recovered in the intestinal segment (jejunum and cecum), which supports the general assumption that microbial material from the cloaca ascends the full length of the oviduct (94). However, the relative abundance of genera composing the microbiota is distinct when comparing the cloaca and vagina to the four other reproductive tissues (93). Similar to the oviduct, Proteobacteria and Firmicutes account for most of the bacteria genera identified in cloaca, but some Actinobacteria are uniquely found in the cloacal segment, which suggests that oviduct secretions exert a selective pressure on ascending microbes by restricting their surface appendages and motility or *via* specific antimicrobial molecules. The relative abundance of each genus also varies due to genetics, physiology and health status of the hen, and parameters linked to housing systems. It is noteworthy that some discrepancies in the published data may arise from the methods used to quantify microbes. Despite this known variability, the chicken reproductive tract is commonly characterized by a relatively high abundance of *Enterococcus* (*Lactobacillus*), *Pseudomonas, Acinetobacter* and *Staphylococcus* (92–94). Similar phyla compositions have been described for other birds including great and blue tits (89). Pathogenic and spoilage bacteria such as *Escherichia coli*, *Shigella*, *Pseudomonas, Listeria, Salmonella*, *Staphylococcus aureus* have been reported in eggshell and oviduct (93, 95, 96).

Egg formation ends with cuticle deposition and expulsion. Microbial communities from the uterus, vagina, and cloaca are mainly represented on the surface of freshly laid eggs (93, 96). However, some genera were found to be absent from the eggshell surface (96), suggesting a selective mechanism during microbiota transfer from the hen to the egg outer surface. The composition of the microbiome and the eggshell cuticle coverage is correlated with the prevalence of pathogenic bacteria, including *Salmonella enterica* Enteritidis, at the surface of the eggshell but also in internal egg components. Survival of commensal microbes on the eggshell is likely to depend on many factors that include their capacity to survive on the eggshell surface, to compete with environmental microbes in attachment to the cuticle, their capacity to transition from an anaerobic to an aerobic environment and their ability to resist dehydration (97, 98).

#### 2.7.2 Horizontal Transmission

In contrast to vertical transmission, horizontal transmission involves contamination of eggs by penetration through the eggshell after oviposition, often *via* contaminated feces. After egg laying, surface microbial pressure may be very high depending on the nature of the incubation environment. The type of the nest/incubation chamber, the ambient humidity and temperature, and nature of parental assistance or not (presence of feather, feces, secretions of the uropygial gland), all influence the establishment of post-lay microbiota on the eggshell surface (99).

##### 2.7.2.1 Nest/Incubating Chamber

There is a large diversity of substrates and materials used by reptiles and wild birds to lay their eggs in or fabricate their nests: grass, roots, wood, bracken, moss, feather, dry mud, and sand, in addition to decaying organic matter. The nature of the nest built by wild birds and its wettability largely contributes to the composition of the egg surface microbiome after laying (100). In contrast, commercial fertilized eggs are handled in large incubators under controlled incubation parameters (surface characteristics, temperature, relative humidity). In commercial hatcheries, the systematic use of disinfection procedures with formaldehyde or chemical alternatives have been shown to reduce bacterial load on the eggshell; however, this does not totally eliminate the eggshell microbiome (101, 102). Moreover, the initial degree of bacterial contamination of the shell is positively correlated with the concentration of bacteria in the air of poultry houses (103, 104).

Some birds (megapodes) directly bury eggs in sand, soil, or decaying organic matter that hosts diverse and rich bacterial communities which differ depending on ecosystem type. Bacterial diversity is usually highest in neutral soils and lower in acidic soils (105). The unusual nesting behavior of megapodes that theoretically increases the risk of egg contamination has resulted in evolutionary adaptations of the eggshell. Eggshell from megapodes contains peculiar structures, which improve hatching success (43, 106). The surface of the megapode eggshell displays nodes similar to those of extinct titanosaur dinosaurs. Pronounced nodular ornamentation is an adaptation to an environment rich in organic acids from their nest mound, which protects the egg surface from chemical etching and leaves the eggshell thickness intact. The internode spaces in both megapode and titanosaur species act as funnels, which concentrate condensed water vapor and channel water through a layer of calcium phosphate that creates a barrier to microbial invasion.

##### 2.7.2.2 Parental Origin (Uropygial Gland, Feather, Skin)

After laying, the microbiota from the parental (male and/or female) feathers also participate in the establishment of the eggshell microbiome. The feather bacterial community is diverse and is essentially composed of Firmicutes and Proteobacteria; sequencing demonstrates that they are closely related to soil- and water-borne bacteria. These results strongly suggest that birds have acquired their feather microbiome from the environment (107). The feather microbiota is also influenced by secretions from the uropygial gland, which is an exocrine gland located dorsally at the base of the tail. It produces antimicrobial and antifungal secretions that maintain feather integrity and hygiene by limiting the prevalence of feather-degrading microbes, including parasites (108). Uropygial secretions contain acidic mucins, neutral lipids, glycolipids, and phospholipids (99, 100), and are spread on plumage and on the epidermal layer of the skin of many birds by preening (9, 100, 109). Spreading of uropygial secretions is assumed to limit the colonization of the egg surface, nest and hen feathers by harmful microorganisms, since these secretions reduce moisture levels on the eggshell, possess antimicrobial properties (107) or promote bacterial species that produce antibacterial secondary metabolites (110). Similar observations have been reported for the brood patch, a patch of featherless skin on the underside of birds, which is in contact with the incubated egg(s). Here the hyperplastic epidermis exhibits a high degree of lipogenesis (111) associated with an increase in trans-epidermal water loss (112). This skin differentiation specifically at this anatomical site is thought to provide moisture and possibly antimicrobial chemicals to the egg during incubation and may contribute a unique microbiome to the egg surface during incubation. Thus, the contribution of the parental microbiota to the eggshell microbiome after laying is diverse and depends on bird species.

A study performed on wild passerine birds found that most of the bacterial communities found on the eggshell surface 12 h to 36 h post-laying were present on the brood patch skin, feathers and nest material, but not the cloaca (113). However, various microbial communities of the cloaca, brood patch skin and feathers are connected with each other and with the nest microbiota (nest lining material and surface soil) (113). The central role played by the parental/female secretions in determining eggshell bacterial communities was also reported for the hoopoes (*Upupa epops)*. Indeed, soon after laying hoopoe hens preen uropygial oil onto the eggshell that is covered by depressions known as crypts, which is assumed to contribute to protection of embryos from pathogens (114). Such examples underline the impact on the eggshell microbiota by multiple factors, which varies depending on the bird species and the prevalence of microbes present in the nesting environment at a given time.

##### 2.7.2.3 Incubation Conditions

In the wild, the first eggs laid may undergo embryonic diapause until the last egg of the clutch has been laid, to avoid asynchrony of hatching (115). Some birds like house sparrows (*Passer domesticus*) use intermittent incubation (115) and cover eggs of the clutch with nest lining during the laying period, to protect their eggs from pathogenic microbes (116). In commercial hatcheries, intermittent incubation (termed SPIDES - Short Periods of Incubation During Egg Storage) is also routinely used to synchronize embryonic development (117). There are only few studies related to the impact of the duration of egg storage prior to incubation on the composition of the eggshell microbiota. Commercially, fertilized chicken eggs are disinfected prior to incubation (95, 101, 102); therefore, data related to this specific question is missing from the voluminous chicken literature. However, there are many articles available on the impact of egg storage on the microbiota of unfertilized table eggs, which are stored under very diverse conditions worldwide depending on the climate, on retailer and consumer practices, and on country-specific legislation (98). Many studies have reported the changes associated with the eggshell microbiota of table eggs with duration and temperature of storage, but also upon geographic area (118) and type of housing system (103, 104, 119, 120). In addition, some conditions have been observed to favor the appearance of yeasts at the surface of eggshell, especially when eggs are stored at refrigerated temperatures. *Candida famata* was identified as the most frequently isolated fungal species throughout egg storage (121).

In contrast, the vast majority of reptiles does not parentally incubate their eggs and bury their eggs in soil or in mounds of decaying vegetation (e.g. crocodilians) (122). Consequently, reptile embryos develop under a microclimate that is completely determined by local hydrologic, climatic and edaphic factors. Studies describing and identifying microbiota of reptile eggs are rare. Yet, both bacteria and fungi have been isolated from nests and eggs (123, 124) and have been shown to reduce hatchling success of sea turtle, caiman, and lizards (125–127). Thus, microbial contamination should constitute a strong selective force in reptile eggs, and selection for antimicrobial mechanisms, including cuticle properties, should be high.

The initiation of incubation/brooding will contribute to the establishment of the composition of the eggshell microbiota. There is increasing evidence that fertilization and incubation dramatically reduce the abundance and diversity of microbial assemblages on eggshells. Moisture increases the occurrence and proliferation of microbes on the eggshell surface; however, incubation facilitates surface drying and limits bacterial growth (41). In pearly-eyed thrasher (*Margarops fuscatus*), unincubated eggs were observed to harbor bacterial and fungal pathogenic microorganisms that grew rapidly on shells of newly laid eggs but declined to undetectable levels when eggs were incubated (128, 129). Incubation also exerts a selective effect on eggshell microbiota, whose composition was shown to fluctuate during incubation in ground nesting passerines (130–132). A similar trend was observed in reed warbler (*Acrocephalus scirpaceus*) (133) and in Eurasian magpie (*Pica pica*) (134). In the latter study, incubation was associated with the growth of harmless bacteria and the suppression of pathogenic bacterial taxa, hence reducing the diversity of the eggshell microbiome.

To conclude, in order to counteract the adverse effects of moisture and the presence of potentially pathogenic microbes in their environment, birds have developed multiple and complementary strategies. To maintain the commensal/beneficial bacteria at the surface of the egg, some avian species have evolved hydrophobic shell surfaces that resist water absorption to limit environmental microbial adhesion (135) while other eggshell structures encountered in hoopoe eggshells (complex cavities known as crypts) favor adhesion of symbiont-carrying uropygial secretion (136). Such strategies have been suggested to be common among oviparous species (137), and may be particularly advantageous to species that lack parental care, such as megapodes. In addition to protecting the developing embryo, the eggshell microbiome may be a vehicle to transfer microorganisms to the progeny and to contribute to the establishment of the gut microbiota of growing chicks after hatch (92, 99, 138).

### 2.8 Role of the Microbiome

The eggshell microbiome can theoretically participate in egg defense *via* multiple indirect and synergistic effects. Most hypotheses of such an activity are based on general mechanisms that have been described in other biological systems. However, compared to many biological systems, it is noteworthy that whereas there is a complex feedback between the microbiota and cellular immunity of the host, such cellular mediators are absent at the acellular eggshell surface.

The first mechanism by which the cuticle microbiota may participate in limiting pathogen establishment is the competition between commensal microbiota and environmental pathogens for adhesion to the eggshell surface. The microbiota from maternal origin may form a biofilm thereby limiting the accessibility of eggshell seeding sites for other microorganisms. Indeed, adhesion has been shown to be critical to understanding competition within microbial communities (139) and it is likely that such a competitive exclusion is occurring at the eggshell surface. A recent study demonstrated that removal of cuticle significantly increased number of adhering *Salmonella* Typhimurium cells on the outer eggshell surface indicating role of cuticle in modulating bacterial adherence (34). The cuticle layer experiences a sequential deposition of microbes, initially from the reproductive tract/cloacal segment and then from the external environment (93, 95, 96). These bacterial communities are thought to form the initial microbiota that will adhere to the eggshell surface and saturate adhesion sites, thereby blocking adhesion of opportunistic pathogens to the eggshell. In certain ecosystems, the formation of biofilms has been shown to confer an advantage to seeding bacteria that exploit the nutritive potential of organic particles that are present locally (140). Indeed, the competition for available nutrients is a potential additional mechanism by which eggshell bacterial core species may remain dominant. Generally, the uptake of nutrients by bacteria is constrained by the size of organic particles and their solubility in water. Therefore, the efficiency of assimilation of nutrients by microorganisms will depend on the secretion of microbial oxidoreductases and hydrolytic enzymes (141) that trigger the breakdown of organic particles to aid assimilation. Some cooperative interactions between microorganisms forming the dominant species may also exist (123), but have not yet been described for the eggshell microbiota. A third mechanism relies on the production of antimicrobial substances or metabolites including acidifying substances (lactic acid, acetic acid, formic acid, etc.) (142) that can act as potentiators of antimicrobial proteins and peptides (143), or antimicrobial molecules (ethanol, fatty acids, hydrogen peroxide and bacteriocins) (142). The presence of these substances in the microbial layer depends on the nature of the organisms characterizing the eggshell microbiome.

## 3 Cuticle Functions Across Egg Laying Species: Reptiles, Avian Species

The eggshell is an essential component of cleidoic eggs that acts as the main interface between embryos and their external environment. The cuticle, together with the eggshell, restrict the movement of water and bacteria through the shell respiratory pores. An intact cuticle functions as the first line of defense against entry of contaminating pathogens. Indeed, in birds, a few comparative studies have shown that cuticles, in particularly those that contain nanospheres, are very effective at decreasing attachment of bacteria to shell surfaces and at preventing bacterial penetration into the egg contents (144).

Cuticles might also provide mechanical protection against damage produced by the incubating parent or by the hard substrate inside the nest. For example, vaterite nanospheres on bird eggshells and thick layers of aragonite on turtle shells can act as very effective shock absorbers (145) and reduce shell erosion in acidic nesting environment (146). Although we have a general knowledge of the function of eggshell cuticles, mechanistically, we know very little about how diversity in cuticle morphology and chemistry affects function, particularly in reptiles, or how environmental pressures shape the relationship between form and function.

### 3.1 Physicochemical Protection

The cuticle affects eggshell wettability, water vapor conductance and regulates ultraviolet reflectance in various ground-nesting species.

#### 3.1.1 Eggshell Wettability/Hydrophobicity

The cuticle layer increases the hydrophobicity of the eggshell surface to prevent wetting and water entry through the shell surface (pores or microcracks) in order to reduce the probability of bacterial infection (42, 147, 148). Removal of cuticle uncovers the hydrophilic eggshell surface, indicating that cuticle proteins are important for the hydrophobicity of the outer surface of the eggshell (34). Contact angle, a measure of surface hydrophobicity, is positively correlated with cuticle chemical components and negatively correlated with the carbonate signal of the underlying shell (34). Studies are ongoing to evaluate the potential correlation of contact angle with cuticle staining. Moreover, an inverse correlation between contact angle and *Salmonella* adherence suggests that surface hydrophobicity, due to the presence of cuticle proteins, can reduce bacterial contamination and promote food safety of table eggs (34).

#### 3.1.2 Water Vapor Conductance

**Figure 8:**
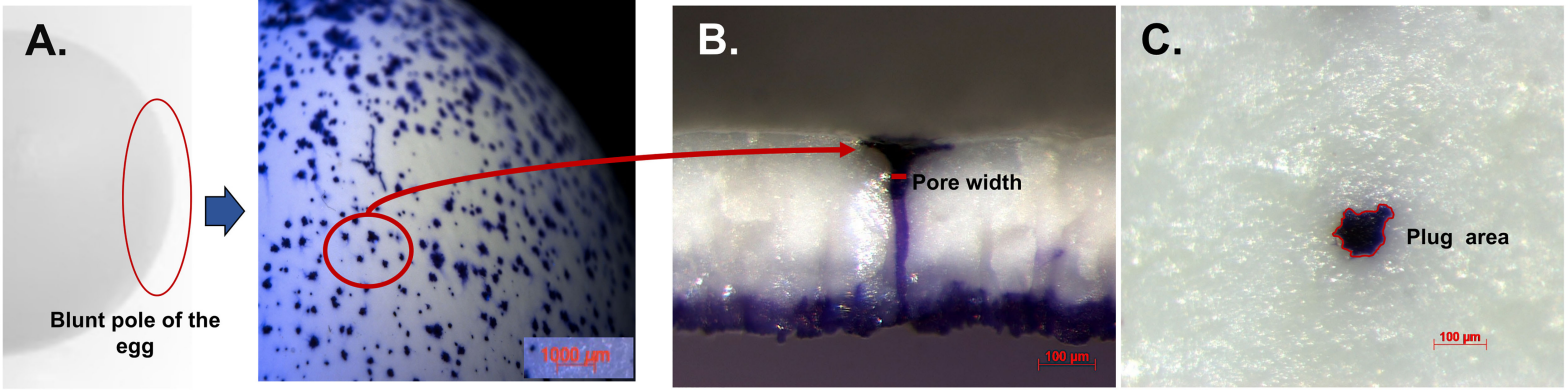
Crystal violet stained cuticle protein in pores and plug visualized by stereomicroscopy. **(A)** Outer surface of white ungraded chicken eggshell (21 wk) showing pore surfaces at 10X. **(B)** Cross-fractured eggshell showing plug and pore lined by stained protein at 150X magnification. **(C)** Outer surface of eggshell showing magnified pore plug at 150X. Reprinted from Poultry Science, Vol. 97, Issue 4, Kulshreshtha et al., Cuticle and pore plug properties in the table egg, 1382-1390, 2018, with permission from Elsevier.

Water is normally lost from the egg during incubation. During the incubation process, an egg must lose sufficient water to create the air cell where the embryo will initiate pulmonary respiration before hatching. For example, the optimal moisture loss (which equals weight loss) is approximately 12-14% until pipping, or on average 0.6% per day in chicken and turkey eggs (149, 150). The diffusion of water vapor from the egg interior to the external environment is dependent on the permeability/porosity of eggshell (69, 144). The eggshell cuticle coats the walls of the pore interior, blocks the funnel-shaped pore mouth as a “pore plug” and covers the eggshell exterior (**Figure 8**), and is a significant regulator to maintenance of water vapor conductance across the shell (151). The rate of water loss is a function of pore number, length and cross-sectional area (34, 151). Cracks and fissures in the cuticle connect the lumen of the pore mouth to the exterior/outer surface of the egg and provides pathways for gaseous diffusion. The fissured cuticle surface contributes to an increased water conductance in chicken eggs (152). Thick eggshell with low density of pores (number of pores/shell surface), or pores with a small cross-sectional area, display low water conductance (151). In chicken eggs, a variable impact of cuticle removal on water vapor conductance was observed (69, 152). Removal of cuticle in eggs from other species such as goose and turkey greatly increased water vapor conductance (69, 151).

In addition, the cuticle also affects gas exchange across eggshells in a mode that is dependent on their thickness and nanostructuring (69, 144). In other species, like the Adélie penguin (*Pygoscelis adeliae*), the cuticle prevents water loss in the severe dry Antarctic environment (137). The cuticle in reptile eggs is assumed to serve a similar function and restrict the passage of water (44, 46). This function might be of particular importance for species with flexible eggshells like snakes, some turtles and lizards (**Figure 4**). Flexible eggshells are composed primarily of proteinaceous fibers, and are inherently more porous relative to fully calcified eggshells. Since porosity governs the movement of gas and liquid between the egg contents and the environment, these flexible shells are highly permeable (153) and therefore could be more prone to desiccation or flooding. Alternatively, hygroscopic organic cuticles could make these flexible eggs more efficient at absorbing water vapor from the environment, potentially allowing them to develop successfully in a larger range of environments, even arid ones.

#### 3.1.3 UV Protection

Optical properties of eggshell and cuticle are important for avian reproduction and influence biological functions such as heating and UV protection (42, 154). The calcified eggshell is an effective UV scattering structure, while the cuticle partly absorbs UV light in a broadband manner. Thus, the cuticle absorbs incident UV radiation, while the underlying calcified shell reflects it with a high scattering efficiency at two different wavelengths (ca. 252 nm and 314 nm in chicken eggshells) (18). Moreover, cuticle pores are responsible for the backscattering peaks observed in the UV range (18). The organic components of the cuticle absorb UV radiation and prevent harmful wavelengths from damaging the embryo and egg interior (42, 154, 155). Cuticle removal from the chicken eggshell surface results in an increase in UV-chroma (the proportion of UV reflectance to the total reflectance), indicating that these organic components selectively absorb wavelengths in the UV range (156). Amino acids of proteins in the eggshell cuticle have distinctive absorption spectra as compared to calcite and absorb maximally in the UV range (156, 157).

#### 3.1.4 Cuticle and Pore Plugs

**Figure 9:**
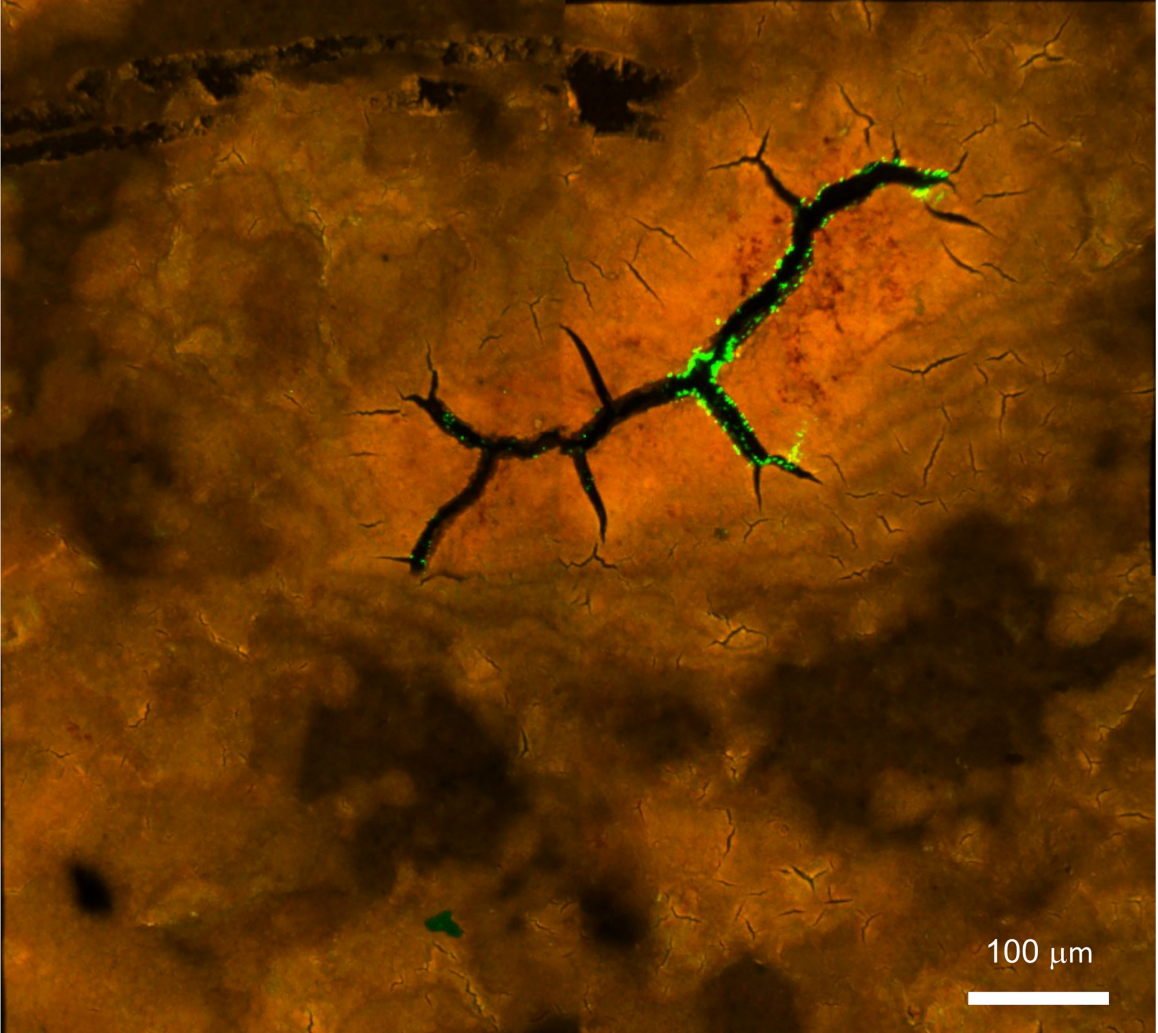
Confocal fluorescent image of *Salmonella* Typhimurium on the outer surface of white ungraded chicken eggshell cuticle. *S. Typhimurium* localized near cracks and fissures of cuticle on the outer surface of ungraded eggshell. Red fluorescence= cuticle protein; Green fluorescence= GFP expressing *S. Typhimurium* cells. (Original figure by GK).

Pathogens can enter the egg interior either though naturally occurring respiratory pores which traverse the eggs or through the micro-cracks in defective eggshells (**Figure 9**).

##### 3.1.4.1 Cuticle Pores

The frequency/density, size and shape of respiratory pores depend on the species (10, 11). In chickens, the pore density varies significantly between different eggshell regions, with approximately twice as many on the blunt pole as compared to sharp pole of the egg (16–18). As assessed by X-ray micro computed tomography (micro-CT), pore diameter varies between different regions of the eggshell (16). Moreover, significantly narrower pores are present at the sharp pole compared to the equatorial region or blunt pole of the egg (16, 17, 158, 159). The presence of incomplete pores which do not span the depth of the shell have also been demonstrated (16). These variations show regional differences in the gas and water permeability across the shell with the blunt pole surrounding the inter-membranal air sac being the principal site of gas exchange during chick embryo development (4, 16). Higher frequency/density of pores at the blunt pole facilitates water loss (approx. 12-14% during incubation) in this region, which is essential for optimum hatchability (4, 160). Functional pore area has been shown to positively correlate with egg weight, size and pore number (16, 17). This positive correlation indicates that avian eggs have evolved to regulate the gas and water exchange required for optimal embryonic development and hatchability by combining these variables. Also, a risk of pathogen invasion sets an upper biological limit on the diameter of eggshell pores (16). Excess water loss in eggs of species that reproduce at high altitude (lower atmospheric pressure) is prevented by a reduction in pore area, not number, which is highly correlated with barometric pressure (11, 160).

##### 3.1.4.2 Cuticle Pore Plugs

**Figure 10:**
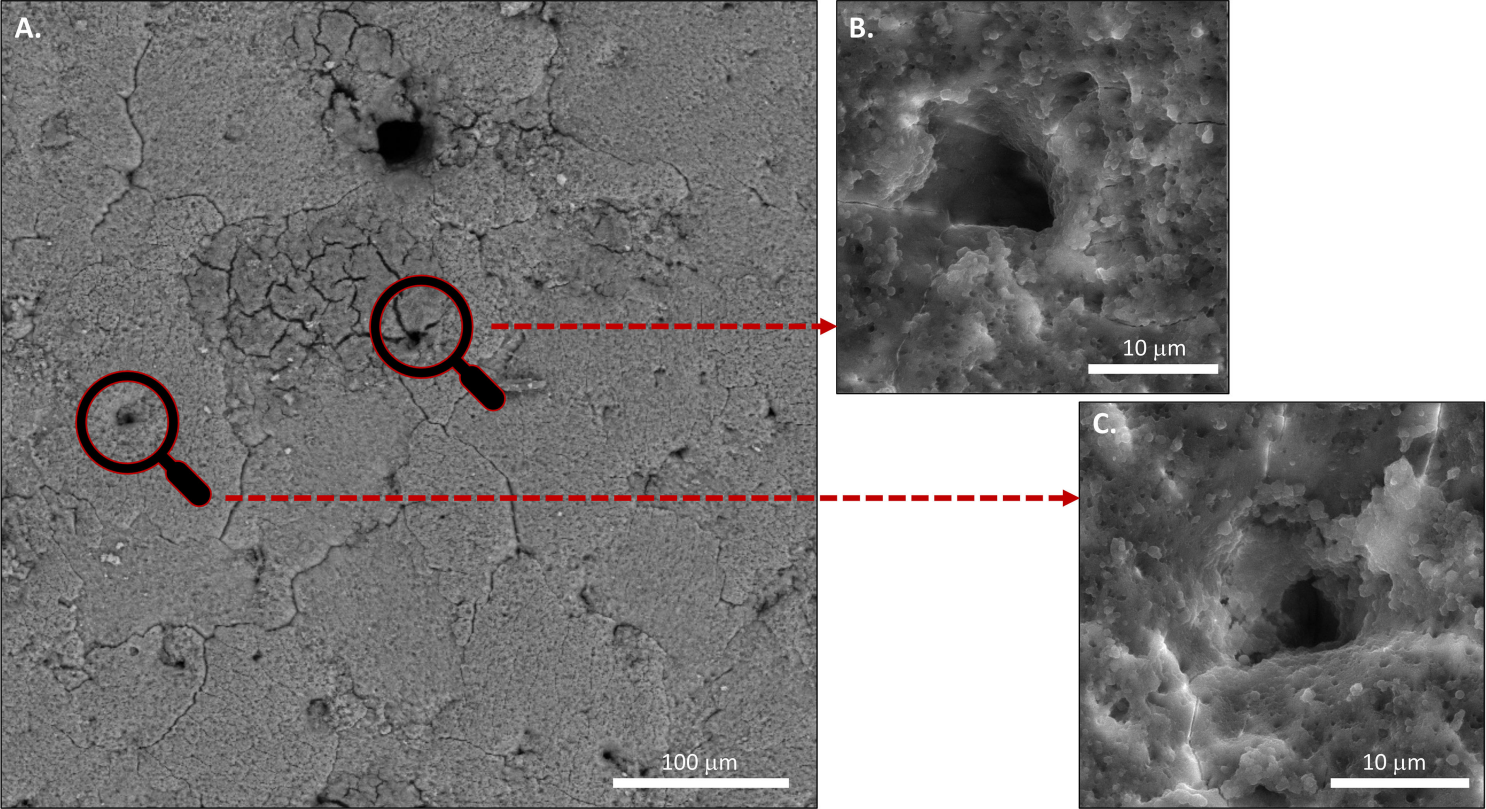
Scanning electron microscopy (SEM) images of **(A)** Outer surface of chicken eggshell showing openings of pores at 1000x. **(B)** and **(C)** at higher magnification 5000X. (Original figure by GK).

The funnel shaped pores of avian eggs are capped with organic spheres called cuticle pore plugs (**Figures 3**, **8**, **10**) (17). Pore plugs are more stable with respect to hen age and egg washing, and compensate for poor cuticle coverage in order to provide protection to the egg against entry of contaminating pathogens *via* the respiratory pores. SEM analysis shows a concave depression on the outer surface of each pore plug, reflecting its continuity with, and origin from, the surface cuticle (**Figure 10**) (17). At oviposition, the cuticle is a thick viscous liquid, which does not mature until approximately 6 h after expulsion (161).

### 3.2 Innate Immune Functions

#### 3.2.1 Cuticle Proteins With Known Biological Functions

The cuticle proteome has been characterized in chicken eggs. A number of them have been identified in various egg components including eggshell matrix and egg white, and characterized using SDS-PAGE, Western blotting, immunofluorescence, colloidal-gold immunocytochemistry and LC/MS/MS proteomic analysis (11, 24, 25, 32, 162). It remains unclear whether the solid-phase, dehydrated nature of the cuticle layer allows these proteins/enzymes to fully express their solution activities.

##### 3.2.1.1 Egg White Proteins

Lysozyme C, Ovalbumin, ovoinhibitor, ovotransferrin, cystatin: These are well-known egg white proteins which are also identified in the cuticle layer; however, their relative abundance in the cuticle is unlike that of egg white (11, 24, 25). For example, in the chicken cuticle, lysozyme is almost 4-fold more abundant than ovotransferrin (25). Lysozyme C is secreted in all segments of hen oviduct especially isthmus and uterus; and displays bactericidal activity by hydrolyzing cell-wall polysaccharides of Gram-positive bacterial pathogens (11, 24, 25, 32, 162). Ovalbumin is expressed in magnum and shell gland and its proteolytic fragments digested with trypsin display antimicrobial activity against *B. subtilis* (25, 163). Ovoinhibitor is a major Kazal-type serine protease inhibitors (SERPINs) secreted in the oviduct. It functions as an anti-protease and exhibits antibacterial action by inhibiting serine proteases including trypsin, chymotrypsin, elastase, as well as subtilisin produced by *Bacillus* spp (25, 164). Ovotransferrin is secreted in magnum, isthmus and uterus and exhibit antimicrobial activity by sequestering iron ions (Fe^3+^) which is essential for growth of Gram-negative bacterial pathogens such as *Salmonella*, *E. coli*, and *Pseudomonas* spp (165–167). Cystatin is a non-glycosylated protein expressed in the oviduct. It targets cysteine proteases and exhibits antimicrobial activity against bacteria, viruses, yeast, and parasites (25, 168). These proteins have been extracted from various egg components, and characterized using SDS-PAGE, Western blotting, immunofluorescence, colloidal-gold immunocytochemistry and LC/MS/MS proteomic analysis (11, 24, 25, 32, 162).

##### 3.2.1.2 Eggshell Specific Proteins

The most abundant cuticle protein is ovocalcyxin-32 (OCX-32), which is encoded by the retinoic acid receptor responder 1(RARRES1) gene, highly expressed in the isthmus and uterine region of hen oviduct, and is secreted by the surface epithelial cells (24, 25, 30). In commercial egg production, RARRES1 is a candidate gene for selection of egg quality traits (169). OCX-32 haplotypes are correlated with eggshell color in white egg lines and line-specific effects have been demonstrated on egg quality parameters such as albumen height, early egg weight and yolk weight (169). Immunofluorescence demonstrated that it is enriched in the cuticle layer and in the outer calcified layer, and Western blotting revealed its presence at high levels in the uterine fluid during the termination phase of egg formation (30). The OCX-32 sequence has 30% homology to a carboxypeptidase A inhibitor, suggesting that it is an antimicrobial protein which could be effective against bacterial proteases (14, 24). Recombinant OCX-32 expressed in *Escherichia coli* exhibited carboxypeptidase A inhibitory activity and inhibited growth of Gram-positive *Bacillus cereus* (170).

Ovocalyxin-36 (OCX-36) is an eggshell-matrix protein which is abundant in eggshell membranes and has been identified in other egg components including vitelline membrane, egg white, and the inner part of the shell. It is secreted in the uterine fluid collected during the calcification step of shell mineralization (4, 8, 171). It is also expressed in hen digestive tract (172). Chicken OCX-36 is encoded by the BPIFB3 gene; its protein sequence shares 20-25% sequence homology to mammalian proteins associated with the innate immune response, such as lipopolysaccharide-binding proteins (LBP), bactericidal permeability-increasing protein (BPI) proteins and palate, lung and nasal epithelium clone (Plunc) family proteins and is involved in lipid binding functions (173, 174). It displays lipopolysaccharide and lipoteichoic acid binding activity, suggesting its role as a pattern recognition molecule, which binds bacterial endotoxins in order to eliminate pathogens such as *Staphylococcus aureus* (31).

Ovocleidins (OC-17, OC-116) are mainly secreted in hen uterine fluid during the active calcification stage of shell formation (14, 168). Ovocleidin-116 (OC-116; MEPE, matrix extracellular phosphoglycoprotein, is the mammalian ortholog) is a major component of the chicken uterine fluid and is the most abundant matrix protein in the eggshell (14, 174). Ovocleidin-17 is a phosphorylated protein with a C-type lectin (CTL) domain, which exhibits bactericidal activity against both Gram-positive and Gram-negative bacteria including *Bacillus subtilis*, *Staphylococcus aureus*, and *Pseudomonas aeruginosa* (175). This protein is dispersed throughout the calcified matrix with greater abundance in the mammillary layer (5). CTL proteins that are homologs of OC-17 have been identified in eggshells of many bird species, and it is thought that OC-17-like/XCA-2 and XCA-1 are eggshell-specific proteins restricted to vertebrates that produce a calcitic shell (174). Phylogenetic analysis indicates that these CTL family members have been duplicated multiple times during avian speciation (176).

### 3.3 Physical vs Chemical Barrier

An intact cuticle forms pore plugs that occlude the respiratory pores and is an effective physical barrier against microbial penetration (5, 17, 28, 29). The pore openings on the surface of the eggshell are covered by the proteinaceous cuticle, which extends into pores up to 50 μm to restrict bacterial entry (29, 177). The pore plugs permit water/gas exchange (35, 177) while physically impeding bacterial passage/penetration and restricting access to the egg interior. In addition to this physical defense, the eggshell cuticle components also function as a chemical barrier. Various cuticle proteins including OCX-32, OCX-36 and lysozyme C possess antimicrobial activity (24–26, 30–33). Moreover, a recent study showed that removal of cuticle using bleach treatment significantly increased the number of adhering *Salmonella* Typhimurium cells on the eggshell surface as compared to those with an intact cuticle (34). This evidence suggests that, in addition to functioning as a barrier to microbial migration through the respiratory pores, the cuticle also functions to reduce bacterial adherence (34).

## 4 Effect of Genetic and Environmental Variables, and Nesting Ecology on Cuticle Evolution/Deposition

### 4.1 Environmental Variables and Nesting Ecology on Cuticle Evolution

Many environmental factors, through their direct influence on the gaseous environment of incubation, or indirectly, e.g. influencing the growth of pathogens that can contaminate the egg, have the potential to affect the presence of cuticle on eggs. A few lines of evidence already suggest that the cuticle is an evolutionarily labile structure that can vary greatly in relation to the nesting environment. Across Sauropsida, the clade that includes all amniotes except mammals, rigid eggshells evolved convergently (178, 179), thus, any external shell component would have also certainly been independently acquired and/or lost. A few decades ago, Board (1982) noted the presence of cuticle on eggs of certain species nesting in wet environments and suggested that these were an adaptation for waterproofing eggs in those nests (10). More recently, D’Alba et al. (42) provided the first comparative study considering the association between the eggshell cuticles and nesting ecology (42). This study suggested that the presence of cuticle is an ancestral trait that has been lost multiple times in birds but conserved in species that nest in hydric environments, where wet incubation sites expose eggs to a higher risk of embryo asphyxiation and microbial infection.

Exposure to solar radiation could also influence the presence of cuticle on eggs, as they prevent harmful ultraviolet wavelengths from reaching the embryo (155, 156). This effect could be particularly important in species that reproduce in exposed nests (birds), which receive more sunlight. In buried eggs, as in most non-avian reptiles, moist and acidic environments may increase the occurrence of eggshell corrosion (146) Thus, the cuticle could be an adaptation to counter this effect. Indeed, eggshells in these types of nests are characterized by cuticular layers (44) However, for the most part, the effect of environmental factors on evolution of cuticle deposition in reptiles is largely unknown, meaning that considerable work will be necessary to test these hypotheses.

### 4.2 Housing System and Cuticle Deposition

Conventional cages are the most common production system for table eggs in many jurisdictions; however, many countries such as the United States and Canada are in the process of supplementing or replacing conventional cages with alternative housing systems such as free-run, free-range and enriched system (180, 181). In Europe, the use of conventional cages for laying hen has been banned since 1 January 2012 (182). This is mainly due to hen welfare concerns, arising from an increased interest in retail egg production systems from the consumers, egg producers, legislators, consumer groups as well as animal welfare organizations (181, 183). Conventional cages provide good health, hygiene, and low mortality, but restrict normal behaviors such as movement, stretching and wing-flapping; however, hen aggression and nesting behavior are modified in cage-free systems (184, 185). Previous study has shown higher cuticle coverage in free range, barn systems and cage eggs (180). This is in agreement with a recent study where better cuticle quality and lower *Salmonella* adherence was observed in eggs from free range systems as compared to enriched and free run (34). This observation could reflect lower bird stress levels in free range systems; however independent stress biomarkers were not measured in this study. Cuticle deposition requires a normal endocrine cascade and is susceptible to environmental stressors (56, 186). Birds under stress release adrenaline, which accelerates the transit time of the egg through the reproductive tract, leading to early termination of cuticle deposition in the shell gland (56, 186). Furthermore, animal handling also affects behavior and productivity in hen production systems (187–189). Poor egg production and quality is more prevalent in farms where the animals are roughly handled and frightened of humans (187, 188) due to the physiological changes associated with elevated stress (188, 190)

### 4.3 Heritability and Genetic Correlation With Cuticle Deposition

Studies in chicken have investigated the genetic contribution to cuticle variability, usually expressed as heritability. In general, the level of heritability is moderate, lying between 0.26 and 0.53 for a range of strains of hen from broiler breeders to layer strains producing both brown and white eggs (27, 85, 191). There was no evidence of negative or positive genetic correlation with production traits; in other words, the genes for determining cuticle deposition were not linked to traits such as egg laying rate, egg weight, eggshell breaking strength, and body weight at laying age etc. (27, 85, 191). This was not the case for the genetic correlation between the cuticle and egg shell color, where a link was observed. This interaction was different between lines and may reflect differences in the relationship between cuticle deposition and pigment deposition, which at least for a commercial brown egg layer, were independent events (55). However, that may not be true in every line. In terms of the Minolta L* value of an egg which measures luminance from light to dark or a* on the green-red axis there was evidence for a significant positive correlation of darkness and redness with cuticle deposition in a Rhode Island red (RIR) line (191). However, when a range of lines with different egg colors was compared there was non-significant genetic correlation in a RIR line between pigment and cuticle deposition, a positive genetic correlation in a white and tinted egg layer and a negative relationship in a white rock brown egg layer (85). So clearly, the relationship between pigment and cuticle varies between lines and may reflect simply differences in the timing and amount of deposition of both cuticle and pigment. Potentially the most troublesome relationship is the positive correlation observed in white egg layers between the Minolta b* value and cuticle deposition, which might result in a yellower and less white appearance when there is more cuticle (85). The most significant genetic correlation is that with hen age, which demonstrates that the cuticle quality measured in early eggs is valid for selection for those laid later in life, and therefore, only one measurement needs to be made (85).

### 4.4 Hen/Flock Age and Cuticle Deposition

Egg quality parameters, such as eggshell color, shell breaking strength, eggshell thickness, etc., decrease with hen age, which determines the end of productive life of a flock (12, 192). Some studies have shown that the thickness of the cuticle is also dependent on hen age and has been reported to decrease with increasing age of the hen (17, 20, 180, 193, 194); however, this aspect remains controversial. Older hens at the end of the laying cycle (about 70 weeks) lay eggs with a lesser amount and patchier distribution of cuticle. This greatly increases eggshell permeability to water and can make eggs more susceptible to trans-shell bacterial contamination (20). Similarly, other studies have confirmed a poor cuticle coverage in eggs from older hens (37, 83). Additionally, many recent studies have also confirmed an age-related decline in cuticle deposition (17, 20, 180, 194). For example, completeness of cuticle coverage was higher in eggs from the 44-week-old flock as compared to 64- or 73-week-old flock (180). Another study validated less cuticle on eggs laid by hens at 60 weeks of age as compared to those from 25-week-old hens (194). These studies used cuticle protein staining to validate an age-related diminishment in cuticle. Furthermore, a combination of both cuticle staining and infrared spectroscopy (ATR-FTIR used for measurement of cuticle chemical composition, see 2.4, 2.6) confirmed that cuticle coverage and chemical composition are dependent on hen age (17, 20). Eggs from younger hens (up to 48-week-old) had better cuticle quality and higher protein signals as compared to older hens (up to 70-week-old) (17). However, previous findings showed that there is no effect of hen age on the cuticle coverage and a greater diversity occurs in cuticle deposition among hens (82). These findings are consistent with a recent study that did not observe an age-related decline in the cuticle coverage. In this study, cuticle deposition was evaluated in hens from 24-50-week of age (81), while most of the previous studies observed an age-related decline beyond 50-week of hen age (17, 20, 180, 194). A major limitation of former studies (17, 20, 194) is that cuticle measurements were not carried out on eggs from the same hens. Observations from different birds from different flocks and at different ages increases a possibility of finding conflicting evidence due to enhanced variability. Future studies where eggs from individual hens are followed for a long duration is recommended to determine if cuticle deposition and its chemical composition decline with age (81). This is particularly important, since laying persistency is a major trait currently being developed further in laying hens. The “long life” layer, which will be capable of producing 500 eggs in a laying cycle of 100 weeks, is on the horizon (192).

### 4.5 Effect of Egg Freshness, and Commercial Washing on Cuticle Stability

There are natural variations in the amount/thickness of cuticle present on eggshell surface and its presence/stability is affected by factors including egg freshness, and commercial washing (17, 20, 27, 83).

#### 4.5.1 Egg Freshness

In freshly laid eggs, the cuticle is soft and has a mucous appearance. It dries within a few minutes after oviposition, but there are longer time changes which cannot be appreciated visually and that affects its functionality (i.e., resistance against bacterial penetration) (20). Therefore, the cuticle of freshly laid eggs (less than 3 h after oviposition) is immature; consequently, it is not able to resist bacterial penetration. Eggs that are between 6 and 72 h old have a fully mature cuticle which is able to resist bacterial penetration more effectively. In contrast, older eggs (after 72 h from oviposition), have a dried cuticle that is less able to impede the movement of water and bacteria through fissures to gain access to the egg interior. Thus, the protection of the egg by the cuticle is only temporary, being optimum in the first days after laying. This maturation and stabilization of the cuticle during this early period improves its structural stability and mechanical properties (73).

#### 4.5.2 Commercial Washing

The practice of washing and sanitization of eggs removes dirt including any debris and reduces microbial load on the eggshell surface, thereby reducing any potential of horizontal transmission as well as the incidence of cross contamination during food handling or preparation (195, 196). Regulatory restrictions on egg washing varies in different countries; for instance, in the United States, Canada, Australia, and Japan, egg washing is a mandatory practice before retail sale (17, 70). In these countries, egg washing is considered safe and is an essential step in the hygienic production of eggs (84). On the other hand, commercial egg washing is not permitted within the European Union, by Regulation (EC) No 589/2008 (197), as it could damage or partially remove the cuticle and cover up poor husbandry and hygiene standards (198). According to the National Food Safety Standards (GB 2749-2015), egg washing is neither mandatory nor prohibited in China; consequently, 95% of Chinese commercial table eggs are unwashed (personal communication, V, Guyonnet). The commercial washing process can remove some of the outer surface cuticle; however, the cuticle plug still remains firmly lodged and continues to block bacteria from entering the respiratory pores (17). Moreover, egg washing procedures do not necessarily damage the cuticle (83). Growing public awareness of food safety issues has changed the consumer perception about egg quality. Their interest has progressed from clean shell and physical properties to microbial resistance of an egg (199). Hence, recent findings demonstrating that commercial washing does not damage the cuticle or pore plug should inspire consumer confidence in the nutritious table egg.

## 5 Conclusions and Way Forward

Studies on origin, synthesis and assessment of cuticle have generated wide interest amongst both scientific communities (i.e. biologists, ecologists) and poultry industry groups. This review identifies challenges and proposes new strategies to better understand innate immune function of the cuticle in a variety of egg-laying species (birds and reptiles), with special focus on the chicken eggshell cuticle. Further research will be necessary to fully characterize eggshell cuticle in relation to the protective function that it plays in innate immunity, for successful reproduction as well as to maintain food safety of table eggs. These challenging topics are summarized in **Table 2**.

**Table 2:** Challenges and proposed new strategies to understand innate immune functions of the eggshell cuticle.

Topic	Current Research	Future Prospects
Characterization of cuticle proteins	Most proteomic studies that have identified and characterized the proteins responsible for the protective capabilities of the cuticle have been conducted in chicken (section 2.4).	-Eggshell cuticle proteomics analysis in a wide gamut of egg-laying species (both avian and reptile) is necessary to provide a better understanding of the link between cuticle properties/coverage and its protective function. -Comparison of cuticle proteome in bird and reptile species is necessary to understand its evolution and response to changes in habitat/environment/climate.
Cuticle quality estimation and characterization of its chemical components	Limited methods are available to measure amount or degree of coverage of the cuticle on eggshell surface in a non-destructive manner (section 2.6). Thus, available methods have limited application in commercial poultry production.	-Cuticle proteins alter the surface hydrophobicity, which can be estimated using contact angle measurements. Cuticle surface hydrophobicity has been negatively correlated with bacterial adherence. -Measurement of contact angle, by adding a droplet of deionized water to the egg surface, could be implemented in a high throughput manner in commercial hatcheries/egg grading systems to evaluate cuticle quality and select/categorize eggs based on hydrophobicity of the eggshell surface.
Cuticle coverage	Patchy distribution of cuticle (section 2.3 and 2.6)	-Cuticle coverage is not complete on surface of the eggshell in some species, while it is absent in other species (section 1 and 2.2). -The mechanism of interaction of microbial pathogens with surface antimicrobial molecules of the cuticle is not well defined. The significance of cuticle completeness in establishing biosecurity of eggs is still not completely understood. It is not clear if cuticle coverage has evolved to sufficiently plug the pores and having uniformly complete cuticle coverage is of less importance in order to protect eggs from pathogens.
Role of eggshell microbiome	Microbiota participate in egg defense by various indirect and synergistic effects (section 2.7 and 2.8)	- Most of these mechanisms are hypothetical and require further experimental study. -It may be difficult to generalize microbiome role to all egg-laying species, considering the diversity of phyla composition that is adapted to substrate modifications (structure/composition of the cuticle) and environmental changes (nesting environment and climate). - The hypothesis that the egg interior is sterile should be experimentally evaluated.
Genetic variation and potential for genetic improvement	A moderate heritability of cuticle deposition is observed in chicken, which is important for genetic progress to increase deposition of cuticle (section 4.3).	-Currently, cuticle quality is not assessed as part of the eggshell quality or egg quality assessment. -Cuticle deposition should be incorporated into breeding programs for egg and meat type birds to reduce vertical transmission or environmental contamination with pathogens in order to improve biosecurity in poultry.
Cuticle pore and plugs	Recent studies have characterized structure of cuticle pores and plugs (section 3.1.4)	-Mechanism of pore formation in eggshell is still not known, the origin of pore and plug during egg formation is still unclear. -Localization of antimicrobial proteins in cuticle pore and plug needs to be evaluated. Elucidating their mode of action will improve methods to maintain egg quality and prevent egg contamination.
-Regulation of synthesis and deposition of cuticle	-Mechanisms controlling cuticle synthesis/secretion are not well-understood (section 2.5).	-Identification and role of potential hormones or cellular signaling cascades that regulate secretion and deposition of cuticle should be investigated.
-Effect of non-genetic factors on cuticle deposition	Stress reduces cuticle deposition (section 2.5). -Effect of age is controversial, with studies showing both no effect and age-related decline of cuticle deposition (section 2.5 and 4.4).	-Studies where eggs from individual hens are followed for a longer duration are necessary to evaluate if cuticle deposition and its chemical composition decline with age. This is particularly relevant to current interest in maintaining flocks for up to 100 weeks of age. -Physiological control of the deposition of cuticle is not well understood.

## Author Contributions

All authors approved the manuscript content and warrant that this review manuscript is not under consideration for publication elsewhere. All coauthors have contributed in a significant way to the final format of the review.

## Funding

This work was supported by funding from the Egg Farmers of Canada (EFC), Livestock Research Innovation Corporation (LRIC) and Canadian Natural Sciences and Engineering Research Council (NSERC, Discovery program RGPIN-2016-04410) to MH. ID was funded by the BBSRC, Lohmann Tierzucht and Aviagen through the BBSRC LINK grants BB/K0070921/1 and BB/K006096/1 ‘Cute-Egg’. The Roslin Institute is funded by a BBSRC Institute strategic program grant BB/P013759/1.

## Conflict of Interest

The authors declare that the research was conducted in the absence of any commercial or financial relationships that could be construed as a potential conflict of interest.

## Publisher’s Note

All claims expressed in this article are solely those of the authors and do not necessarily represent those of their affiliated organizations, or those of the publisher, the editors and the reviewers. Any product that may be evaluated in this article, or claim that may be made by its manufacturer, is not guaranteed or endorsed by the publisher.
